# Emerging role of tumor cell plasticity in modifying therapeutic response

**DOI:** 10.1038/s41392-020-00313-5

**Published:** 2020-10-07

**Authors:** Siyuan Qin, Jingwen Jiang, Yi Lu, Edouard C. Nice, Canhua Huang, Jian Zhang, Weifeng He

**Affiliations:** 1 State Key Laboratory of Biotherapy and Cancer Center, West China Hospital, and West China School of Basic Medical Sciences and Forensic Medicine, Sichuan University, and Collaborative Innovation Center for Biotherapy, 610041 Chengdu, People’s Republic of China; 2 School of Medicine, Southern University of Science and Technology Shenzhen, Shenzhen, Guangdong 518055 People’s Republic of China; 3 Guangdong Provincial Key Laboratory of Cell Microenvironment and Disease Research, Shenzhen, Guangdong People’s Republic of China; 4 Department of Biochemistry and Molecular Biology, Monash University, Clayton, VIC Australia; 5 School of Basic Medical Sciences, Chengdu University of Traditional Chinese Medicine, 1166 Liutai Road, 611137 Chengdu, People’s Republic of China; 6 State Key Laboratory of Trauma, Burn and Combined Injury, Institute of Burn Research, Southwest Hospital, Third Military Medical University (Army Medical University), Chongqing, People’s Republic of China; 7 Chongqing Key Laboratory for Disease Proteomics, Chongqing, People’s Republic of China

**Keywords:** Cancer therapy, Cancer therapy

## Abstract

Resistance to cancer therapy is a major barrier to cancer management. Conventional views have proposed that acquisition of resistance may result from genetic mutations. However, accumulating evidence implicates a key role of non-mutational resistance mechanisms underlying drug tolerance, the latter of which is the focus that will be discussed here. Such non-mutational processes are largely driven by tumor cell plasticity, which renders tumor cells insusceptible to the drug-targeted pathway, thereby facilitating the tumor cell survival and growth. The concept of tumor cell plasticity highlights the significance of re-activation of developmental programs that are closely correlated with epithelial–mesenchymal transition, acquisition properties of cancer stem cells, and trans-differentiation potential during drug exposure. From observations in various cancers, this concept provides an opportunity for investigating the nature of anticancer drug resistance. Over the years, our understanding of the emerging role of phenotype switching in modifying therapeutic response has considerably increased. This expanded knowledge of tumor cell plasticity contributes to developing novel therapeutic strategies or combination therapy regimens using available anticancer drugs, which are likely to improve patient outcomes in clinical practice.

## Introduction

The rapid development of novel therapeutic strategies, represented by targeted therapy, has made great contributions to the improvement of clinical outcomes in patients with cancer.^1,2^ However, such improvements have not been translated into complete remission (CR) due to the inevitable emergence of drug resistance, which is regarded as a major impediment in clinics for achieving complete cures.^1,3^ For decades, along with the identification of various resistance-conferring mutations, researchers have theorized that this therapeutic failure is mainly attributable to genomic mechanisms, such as the acquisition of mutations that occur on the drug target, thus impairing the drug binding and mutation-induced continuous activation of pro-survival pathways.^4,5^ This would suggest that reagents designed to selectively repress such *bona fide* resistance mechanisms hold great promise for the realization of long-term curative effects and the improvement of living quality in patients with cancer. However, drug resistance frequently occurs and remains a clinical challenge.^6,7^ The development of secondary mutations may also provide a mechanistic explanation for such resistance, and may even present a treatment option for patients (e.g., the so-called “next-generation” tyrosine kinase inhibitor [TKI] for non-small cell lung cancer [NSCLC] patients).^8^ The observation that clones with resistance-conferring mutations can pre-exist within an individual tumor prior to drug exposure and be further selected during treatment indicates that merely targeting the validated genetic resistance mechanisms is not enough.^9–12^

**Fig. 1:**
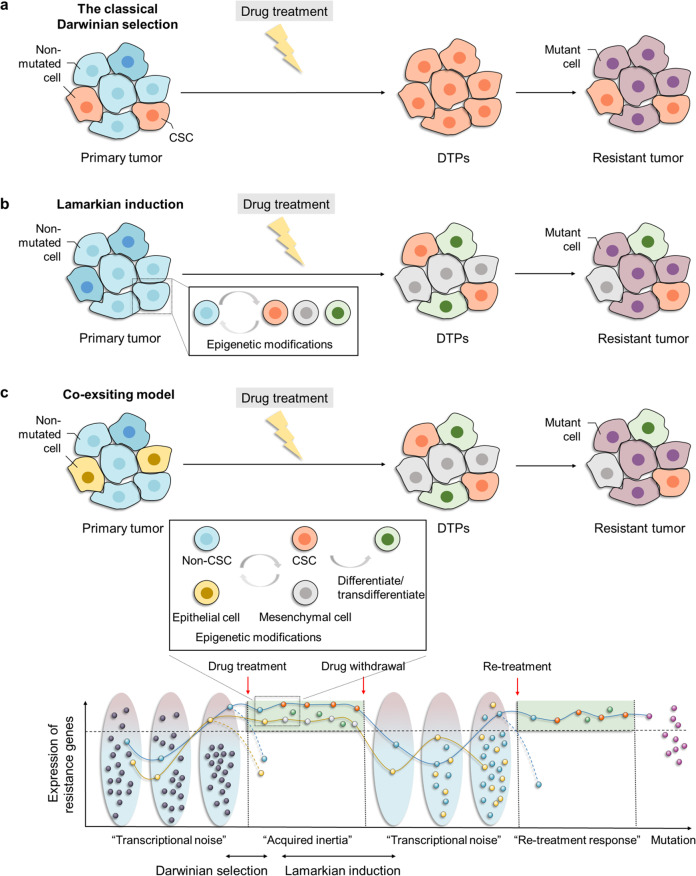
The genesis of DTPs according to natural selection theory (classical Darwinian selection), the Lamarckian induction concept, and the coexisting model. **a** The natural selection theory shows that the preexisting DTPs, here represented by CSCs, can be selected and enriched upon drug exposure. **b** The concept of Lamarckian induction attaches importance to the natural aptitude of tumor cells in adapting to pharmacologic interventions through different levels of epigenetic modifications, giving rise to the emergence and coexistence of DTPs in varying tolerant states. **c** The coexisting model suggests the dynamic transcriptional fluctuation at a single-cell level of resistance-related markers (“transcriptional noise”). A small fraction of tumor cells, whose expression of these resistance-related genes exceeds a certain threshold at the moment of treatment, can survive and be selected (the blue and yellow dot), marking a return to classical Darwinian selection. However, with increasing duration of drug exposure, such a stochastic, transient, fluctuated “survival mode” arrives at drug-refractory state through epigenetic modifications, ultimately resulting in the establishment of a DTP pool. These alterations in the epigenome, which can be summed up as “acquired inertia,” are in agreement with the concept of Lamarckian induction. The solid line represents the changes of resistance-related markers expression with treatment, while the dotted line represents those without treatment (below). CSC cancer stem cell, DTPs drug-tolerant persisters

Occurring in parallel are numerable cases that are not related to genomic/genetic alterations, raising the possibility of non-mutational mechanisms involved in maintaining cancer cell survival and growth upon treatment.^13–16^ For instance, a rare subpopulation of cancer stem cells (CSCs), or poorly differentiated cancer cells equipped with enhanced drug efflux properties and heightened self-renewal potential, is intrinsically more refractory to multiple cancer therapies, suggesting a fundamental role of CSCs as a reservoir for tumor recurrence.^17^ Indeed, such stem cell-like phenotype-dependent relapses have been previously described in patients with chronic myelogenous leukemia following imatinib mesylate treatment^18,19^ and have been further documented in various types of solid tumors.^20–22^ Being regarded as the source of non-mutational resistance, this subpopulation—named drug-tolerant persisters (DTPs), has been widely recognized for its dormant, slow-cycling state and stem-like signature.^13^ Such a so-called quiescent condition of DTPs allows them to survive for long periods of time (weeks to months) in the time frame between being killed and developing mutations.^13^ This window of opportunity seems essential for DTPs—or at least parts of DTPs, to acquire mutation-driven resistance mechanisms by which they can evolve into clinically relevant drug-resistant cells.^23,24^ As such, the tolerance/dormancy/persistence state, which is accepted as an alternative route for acquiring resistance, tends to serve as a “bridge” to link the non-mutational mechanisms with *bona fide* resistance mechanisms (i.e., to connect phenotype-dependent DTPs with genotype-dependent resistant cells^24,25^) (Fig. 1).

Despite knowing the significant contributions made by DTPs to both non-mutational and mutational processes during resistance, controversies still exist concerning the genesis of DTPs between the natural selection theory (classical Darwinian selection), Lamarckian induction concept, and the coexisting model, as described below^26^ (Fig. 1). The natural selection theory is a simple and intuitive principle. Specifically, DTPs, here represented by CSCs in an inconspicuous but preexisting form which are hidden by the overwhelming number of non-CSCs, can be selected and enriched upon drug exposure^17,27^ (Fig. 1a). This theory, based on phenotypic behavior, can also be interpreted as a process for selecting the pre-existing slow-cycling cells under treatment, for example, pre-existing JARID1B-expressing melanoma cells or ZEB2-expressing colorectal cancer cells.^28,29^

In contrast to the “passive” mode of Darwinian selection, the concept of Lamarckian induction attaches importance to the natural aptitude of tumor cells in adapting to internal or external stimuli actively, especially in response to pharmacologic interventions, essentially a concept of therapy-triggered “adaptation” (Fig. 1b). This adaptation, rather than the “one mutation, one outcome” dualistic model, is predominantly reflected in the dynamic change of a number of resistance-related genes through epigenetic events during treatment, laying a mechanistic foundation for the emergence and coexistence of DTPs in varying tolerant states^26,30^ (Fig. 1b). Among the resistance-related markers, the well-characterized drug efflux pump—multidrug resistance protein-1 (MDR1), serves as an example.^31^ In this case, a quick and robust response to vincristine exposure manifesting as phenotypic switching from a low- to high-efflux state, has been observed, which is proved to be a direct consequence of “active” *MDR1* induction via single-cell longitudinal surveillance.^31^ More importantly, once such an induction is triggered, transcriptomic alterations tend to persist for a relatively long time after drug withdrawal^31^ termed “epigenetic memory.”^32^ This is in accordance with the notion that DTPs can transiently evade treatment and maintain the pro-survival phenotype or transcriptome alterations for some time.^12,26^

In actual fact, the dynamic transcriptional fluctuation of resistance-related markers at a single-cell level is more likely to occur before the addition of drug in a manner similar to the so-called “transcriptional noise,” thus giving rise to an incremental source of transcriptional variability for drug selection^16,32–35^ (Fig. 1c). As a result, a small fraction of tumor cells, whose expression of these resistance-related genes exceeds a certain threshold at the moment of treatment, can survive or be selected.^16^ The “internal noise” (e.g., random pattern of transcriptional variability on resistance-related genes) can be viewed as a loaded “weapon” within the “arsenal” of tumor cells to cope with “external noise”^36^ (e.g., drug exposure), marking a return to classical Darwinian selection (Fig. 1c). However, with increasing duration of drug exposure, such a stochastic, transient, fluctuated “survival mode” develops into an adaptive, stable, dormant, drug-refractory state through epigenetic modifications, ultimately resulting in the establishment of a pool of DTPs.^16,34^ These alterations in the epigenome (i.e., “adapting to shape change instead of being shaped”) are in agreement with the concept of Lamarckian induction^31^ (Fig. 1c). Hence, throughout the entire process of the emergence and maintenance of DTPs, these two concepts are not opposite, but rather intertwined and complementary to each other (Fig. 1c).

If one regards the profound transcriptional variability^16^ as the “innate skill” of tumor cells to pursue greater phenotypic diversity, the epigenome-associated dormant state caused by long-term treatment will be more likely the “acquired inertia” of DTPs due to the assumption that the survival skills, that is, overexpression of resistance-related genes, have been gained from the cells surviving initial therapy. This raises the question of “when treatment is discontinued, will the ‘acquired inertia’ fade away and/or ‘innate ability’ be restored?” Consistent with in vitro laboratory experiments, the so-called ‘re-treatment response’’ phenomenon observed clinically supports the occurrence of a reversible process from acquired drug-refractory to initial drug-susceptible state following drug withdrawal^37^ (Fig. 1c). Specifically, a significant fraction of patients with NSCLC who have been through a failed treatment with epidermal growth factor receptor (EGFR)-TKI-based therapy (gefitinib) can immediately achieve remarkable tumor regression following re-treatment with gefitinib after a drug-free interval, demonstrating a second response “window” to treatment with TKIs.^37,38^ Similar re-treatment responses in different cancer types have also been observed with other anticancer agents, including daratumumab,^39^ trastuzumab,^40^ radium-223,^41^ and pembrolizumab.^42,43^ The prerequisite for such a secondary response is that the timing of re-treatment needs to precede the presence of a novel resistance-conferring mutation in DTPs. This can be interpreted as a process of residual DTPs getting rid of the “acquired inertia” while re-activating the “innate skill” or, put another way, a transition from a slow-cycling, drug-refractory to a fast-cycling, drug-susceptible phenotype (Fig. 1c).

Indeed, this reversible phenotype switching, at first glance, can be attributed to the proactive behavioral “changes” of tumor cells to adapt to environmental “changes” albeit in an uncontrollable manner. This also implies that hijacking the mechanisms underlying these “changes” for therapeutic purposes, transforming such a process from uncontrolled to controlled, could be a promising approach. For this reason, studies revolving around the complicated cellular mechanisms involved in the “war” of “hide (phenotype switching)-and-seek (cancer therapy)^44^” have gained increasing prominence in recent years.

In terms of phenotype switching, cell plasticity (the fundamental ability of cells to change their properties in a reversible way actively or passively) plays a prominent role in postinjury tissue repair and regeneration, as well as the restoration of disrupted homeostasis.^45–47^ Besides making contributions to such physiological processes, when activated aberrantly, cell plasticity is involved in the evolution and progression of multiple diseases, particularly cancer.^46–49^ This sheds new light on the explanation of the intratumoral heterogeneity of phenotypic features of cancer during which tumor cells exhibit varying degrees of phenotypic interconversion between drug-susceptible and drug-refractory states.^50^ The above general description of phenotype switching in cases of drug exposure or drug withdrawal represents a universally applicable model of tumor cell plasticity, regardless of what types of cancer are treated or what kind of therapies are employed. However, behind this universally plastic behavior, there exist differences in exactly how cancer cells evade therapy including epithelial–mesenchymal transition (EMT), acquiring properties of CSCs or trans-differentiation potential^26,47,51–54^ (Fig. 1c). Intriguingly, these somewhat functionally overlapping processes are more or less associated with the aberrant (re-)activation of developmental programs, suggesting that similar molecular mechanisms underlying plasticity-driven resistance to therapy may be involved.^55,56^

In summary, in this review, we present a comprehensive description of tumor cell plasticity in response to treatment of various cancers with respect to targeted therapies, chemotherapy, and immunotherapy, and will highlight the mechanisms involved.

## Epithelial–mesenchymal transition (EMT)

The programs of EMT and its inverse process, mesenchymal-to-epithelial transition (MET), are involved in governing vertebrate embryonic development in a highly dynamic, transitory and reversible manner, representing a prime example of cell plasticity, both in normal and neoplastic cells.^55,57,58^ At conceptual and morphological levels, cells undergoing EMT are characterized by loss of apical–basal polarity and the disruption of cell–cell contacts, including tight (e.g., ZO-1), adherens (e.g., E­-cadherin), and gap junctions (e.g., connexins), while acquiring the front–rear polarity and dramatic remodeling of the cytoskeleton organization. This ultimately results in the morphotype switching from “cobblestone-like” shapes to “fibroblast-like” (e.g., vimentin) forms.^55,59–61^ Mechanistically, this process is generally performed by several EMT‑inducing transcription factors (EMT-TFs), such as Snail, zinc-finger E-box-binding (Zeb), and basic helix–loop–helix TFs, and noncoding microRNAs (miRNAs), epigenetic, and post-translational regulators, as well as alterative splicing factors, which are further integrated and controlled by multiple signaling pathways, such as the transforming growth factor-β (TGF-β), wingless/integrated (Wnt), Notch, and Ras-mitogen-activated protein kinase (Ras-MAPK) pathways, in response to paracrine and autocrine stimuli^62–64^ (Fig. 3a). Notably, the EMT-TFs are orchestrated and dynamically regulated themselves by each other and/or other factors in every step of EMT programming, in particular, the two well-established double-negative feedback loops, miR-34/*Snail1* and miR-200/*Zeb* (Fig. 3a). The former regulatory circuit preferentially participates in the initial phase of EMT induction in epithelial cells, while the latter tends to be involved in the development and maintenance of the mesenchymal state.^65–71^ Functionally, it is generally recognized that the EMT programs not only play an irreplaceable role in multiple physiological processes throughout the whole course of an individual’s life, especially during embryonic development (tissue morphogenesis and organogenesis), wound healing, tissue repair, and the induction of pluripotency, but also contribute to various pathological events, including formation of fibrosis and tumor malignancy—from its genesis to development.^59,72–75^

### EMT in carcinoma progression

#### From embryonic development (physiology) to cancer progression (pathology)

Before discussing the impacts of EMT programs on carcinoma progression, it is necessary to mention the inspirations provided by the considerable amount of theoretical and experimental studies on their physiological roles. To be precise, in case of embryonic development, several sequential cycles of EMT and MET—termed as primary, secondary, and tertiary EMT, are highly organized and carefully orchestrated according to separate biological requirements, resulting in the terminal differentiation of specialized cell types and the organization of the extremely intricate three-dimensional (3D) structure of internal organs.^62,74,75^ A typical exemplar is the formation of embryonic heart during which all three cycles are shown to be experienced successively.^62,74,76^ This process is also characterized by the fact that EMT programs take place in well-differentiated epithelia, laying a theoretical and realistic foundation for the occurrence of EMT in other well-differentiated epithelia, including tumor cells. In addition, during the process of wound healing, keratinocytes residing at the wound edge initiate part of the EMT programming autonomously, which leads to the acquisition of an intermediate phenotype—also described as the “metastable” state, along with the capability of migrating towards the middle regions to seal the wound.^73,74,77,78^ Such a functional conversion from stationary to migratory phenotype of keratinocytes, when mapped to cancer progression, denotes that the influence of EMT on the biological behavior of carcinoma cells may be primarily embodied in their ability to migrate or invade—or, even more evocatively, in tipping the scale of “Go (migration) or Grow (proliferation)” towards the “Go/migration,”^79^ which perhaps foreshadows a more aggressive phenotype and a higher metastatic potential of tumor cells.

#### From “complete” to “partial”: the perfect paradigm for tumor cell plasticity

Not surprisingly, the occurrence, performance, and potential roles of EMT in carcinoma cells, as proposed theoretically, have already been determined through compelling experimental evidence in the past two decades, although contradictory opinions exist.^80,81^ These anomalies have stemmed from a lack of convincing evidence at the surgical pathological level to support the concept that EMT is involved in the onset and development of cancers, resulting in a noticeable mismatch of results between laboratory models and human tissue sections.^80,81^ Faced with this mismatch, researchers have provided an appropriate explanation that, in the vast majority of cases of cancer, the induction of EMT may not be as straightforward as it seems—that is, not strictly abiding by the binary-based “all or nothing” principle, but rather a complicated multistage process containing one or more intermediate phenotype(s) with a varying degree(s) of EMT—currently known as the “partial EMT(s)” state.^82,83^ Moreover, a complementary explanation for this mismatch is the indistinguishable expression of EMT markers that results from the coexistence of carcinoma cells with tissue-resident stroma-associated cells inside and around individual tumors, the latter of which can naturally exhibit variable levels of the mesenchymal signature.^84^ Therefore, it seems neither accurate nor objective to measure the “partial EMT” within clinical cancer tissues by the standards of the “complete” one. More importantly, this manifestation of EMT in human cancers echoes the aforementioned “metastable” state in wound healing and the reversible process in renal fibrosis,^85,86^ all of which points to the conclusion that the incomplete activation state appears to be an essential trait of EMT programs, not just during carcinoma progression.

Since the introduction and recognition of the “partial EMT” concept, it should be pointed out that understanding the true meaning of “partial” is the key issue to unlocking the secrets of EMT programs in cancer. As its name implies, the “partial EMT” concept in carcinoma cells can be interpreted simply as a hybrid epithelial and mesenchymal phenotype, existing in the form of clusters and even single cells.^87–92^ Such formats endow cancer cells with the possibility of accessing multifunctional cell clusters and multi-identity single cells so that they can readily cope with the changeable environments.^91^ It is this strong ability of self-adaptation and self-adjustment in tumor cells that represents a perfect mechanism for plasticity, but an enormous threat to cancer patients. Various attempts have already been made in the field to address this experimentally. The ideal approach, proposed by Weinberg,^81^ is to trace the dynamic changes of cancer cells at both an individual and multicellular cluster level, from the time they depart the primary tumor until the point at which metastatic colonization is detectable clinically at a distant organ. In contrast to the initiation (primary tumor) or termination (metastatic tumor) phases, effective monitoring for the intermediate phase (e.g., by using circulating tumor cells, CTCs) is of fundamental importance in exploring the exact role of “partial EMT” in human cancer. However, this is technically challenging due to their very low abundance in blood.^93^

To address this issue, Yu et al.^94^ have developed a quantifiable, dual-colorimetric RNA–in situ hybridization approach to investigate the contributions made by EMT to primary tumors, tumor-infiltrating lymphocytes (TILs), and CTCs from patients suffering from metastatic breast cancer. Compared to primary tumors where EMT occurs very rarely, a significant fraction of patient-derived CTCs display obvious mesenchymal features, the level and quantity of which are positively related to disease progression during anticancer drug treatment.^94^ Further evidence has been obtained through long-term longitudinal surveillance of EMT traits in CTCs from an index patient, whose blood samples were collected serially.^94^ The results from this serial monitoring demonstrate that dynamic changes in the ratio between epithelial and mesenchymal phenotype in CTCs may largely determine the final clinical outcome, that is, response or resistance, both to targeted therapy and chemotherapy.^94^ Similarly, using single-cell RNA-sequencing (scRNA-seq), a subsequent study found direct evidence that the partial EMT program of head and neck squamous cell carcinoma may serve as a valid, independent predictor for adverse clinicopathologic features and malignant biological behaviors, particularly nodal metastasis, through comparative analyses of primary and metastatic specimens.^90^ Together, these observations on clinical samples are in agreement with those from cultured cells and animal models that have tightly linked “partial EMT” to cancer progression, in which therapeutic resistance and metastatic potential are shown to be the closely linked, and extremely threatening.

More recently, with the establishment of an effective, rapid, large-scale single-cell resolution 3D (LSR-3D) imaging protocol capable of visualizing the cellular organization of an entire mammary tumor, Rios et al.^95^ discovered that epithelial and mesenchymal subsets coexist within the same clone in most observable cases of *Pten*/*Trp53* deletion models, offering solid evidence that the induction of partial EMT acts as a ubiquitous adjusting and controlling mode at a clonal level. This finding, from a space perspective, highlights that the induction of partial EMT is not confined to the traditional concept that whether or not carcinoma cells undergo EMT is determined by their localities within an individual tumor, but is more likely an inherent property of most clones wherever they reside. This adds a twist to the traditional view that EMT usually occurs along the invasive front.^92,95^ From a temporal perspective, there seem to be no specific “timestamps” indicating when the partial EMT state of carcinoma cells first occurs as it is seen throughout the period of LSR-3D imaging, including clones from an *Elf5*-driven tumor at its early stage.^95^ This visual evidence ties in closely with data from previous in vivo or in vitro experimental studies, which showed that prior to the development of a malignant phenotype, EMT programs have already started imperceptibly in certain types of human carcinomas, including breast,^96–98^ pancreatic,^99^ and prostate cancer (PCa).^100^ This is in accord with perplexing clinical observations of early metastatic dissemination before the formation of a detectably localized tumor,^101–104^ and the preresistance state of a minor subpopulation of tumor cells prior to drug exposure.^21,22^ The temporal mode of EMT may provide a plausible mechanism by which the above paradoxes can be, at least partially, explained.

Taken collectively, the progressive notion arising from both spatial and temporal perspectives has brought the concept of EMT into a new level of complexity and universality. These two properties can be simultaneously embodied in that carcinoma cells with varying degrees of EMT localizing randomly within an individual tumor, display their respective functional attributes of each clone, or even single cell, ranging from atypical hyperplasia to late-stage metastasis and/or therapeutic resistance. All this suggests that EMT programming during the process of cancer progression is a perfect paradigm for investigating the nature of tumor cell plasticity.

#### From metastasis to resistance

Indeed, from the observations mentioned above, as well as other studies, the proposition that EMT may act as the main driver of metastatic process, drug resistance, maintenance of stemness, and immunosuppression seems justified^55,105–107^ (Fig. 3a). Unfortunately, quite a few patients have metastatic diseases at initial diagnosis, especially in regions where regular health checks and screening are not routine.^108^ There is ample evidence to provide support for the major role of EMT in all steps of the “invasion-metastasis cascade.”^109^ However, the existence of a causal relationship between them remains a long-standing subject of dispute.^110^

Specifically, the contributions made by EMT to metastasis was initially proposed due to the demonstration that inhibiting the expression of Twist and the resulting EMT could significantly alleviate pulmonary metastasis of highly metastatic mammary carcinoma cells in vivo.^111^ Subsequently, similar biological impacts on invasion and metastasis induced by other key EMT-TFs, such as Snail1,^112,113^ Slug,^114–116^ and ZEB1,^117^ were extensively documented in different types of carcinomas. In fact, underlying such functional similarities, these EMT-TFs appear to specialize in handling their precise biological subfunctions in a tissue (spatial)- and/or clinical-phase (temporal)-specific manner; that is, they are organized in a way that tends to be coordinated and complementary, but not redundant.^63^ For example, using a mutant *KRAS* and *p53* driven (KPC) mouse model of pancreatic ductal adenocarcinoma (PDAC), Krebs et al.^117^ have demonstrated that depletion of the EMT-TF *Zeb1*, but not *Snail* or *Twist*,^118^ markedly inhibited PDAC progression from its genesis to advanced metastatic disease. By contrast, using the MMTV-PyMT spontaneous breast cancer model that carries wild-type *TP53*, obvious inhibitory effects on the self-renewal capacity and metastatic potential were observed following *Snail1* excision,^119^ rather than by downregulating *Zeb1*/*2* via forced expression of miR-200.^118^ These repressions can be explicitly reversed in vivo by transient overexpression of *Snail1.*^120^ When connecting these conflicting findings to the aforementioned observations of the contribution made by Twist to breast cancer metastasis,^111,121^ it can be safely concluded that the expression pattern and regulative mechanism of an individual EMT-TF depend critically on the site where the primary tumor occurs, as demonstrated by the roles of *Zeb1* in PDAC,^117^ *Snail1* and *Twist1* in breast cancer,^111,119–122^ and *Zeb1*/*2* in melanoma.^123,124^

Besides differences in the expression of EMT-TFs among cancer types, there also exist differences within each cancer type, represented by the spatiotemporal, synergistic effects of Snail1/Twist1-mediated EMT programs on the promotion of breast cancer progression, especially during metastasis.^125^ This parallel-cooperative functioning mode is reflected by the realization that transitory activated Snail1 plays an indispensable role in EMT initiation, while Twist1 is mainly responsible for the maintenance of late-stage EMT programming, echoing their physiological roles during *Drosophila* mesoderm development.^120,125–127^ Besides functioning synergistically as described above, biological influences exerted by interactions between EMT-TFs vary from one type of cancer to another, and can even perform antagonistically, as shown by the contrasting behaviors of Zeb1 and Zeb2 during initiation and metastatic progression of melanoma.^123,124,128^ Based on this, when mapping the motile, invasive, and dedifferentiated traits acquired through EMT programming to the multistep process of “invasion-metastasis cascade” in the context of mammary carcinoma, it can be hypothesized that Snail-induced initial EMT is associated with early dissemination of carcinoma cells, including local invasion and subsequent intravasation into blood circulation, whereas Twist1-triggered late EMT tends to take place during extravasation and the formation of dormant, scattered micrometastases due to the overlapping functions between EMT and CSC.^55,125,129^ Such a dormant, growth-arrested state, persisting for months or even years, indicates that micrometastatic clusters or single disseminated tumor cells (DTCs) can tilt their functional balance towards stem-like attributes—referred to as “tumor-initiating CSCs,” from a previous migratory phenotype—termed “migratory CSCs’’,^129^ in order to survive in, and adapt to, a distant, unfamiliar microenvironment, serving as potential initiators of macroscopic metastatic lesions.^129,130^ Starting from this concept, re-initiation of tumor growth in a foreign tissue would require maintenance of the self-renewal capacity of DTCs through asymmetric division while differentiating into non-CSCs to spawn fast-cycling epithelial progeny, ultimately giving rise to overt metastases (also called colonization).^129^ Although the mechanism of metastatic colonization shows organ preference,^131,132^ stemming largely from the organ-specific premetastatic niches (PMNs),^133,134^ the restoration of epithelial characteristics, induced presumably by a process of reversible EMT–MET, seems to be a common feature shared by multiple cancer types during seeding of a secondary tumor, only by which means can the EMT-induced invasive phenotype be functionally equivalent to metastatic potential.^59,129,135–143^ With MET programs enabled, macroscopic metastases therefore exhibit the same histopathological trait of epithelial cell predominance as that of their corresponding primary carcinomas, while reconstructing the typical lineage hierarchy between CSCs and non-CSCs lost during EMT induction, as if EMTs had not actually occurred.^84,129,144^ However, many believe that it is such inherent plasticity that ultimately leads to a lack of persuasive evidence at the pathological level to support the essential role of EMT in metastasis, leading to the controversy.^80,81^

Taking all this into consideration, although having yielded conflicting evidence on whether the EMT programs contribute to metastases, the spatiotemporal regulation, and pleiotropic, non-redundant functions of EMT-TFs, the dynamic, transient, and reversible EMT–MET operating system, as well as the extended concept of EMT from dualism (a complete form) to pluralism (multilayered, partial forms), goes a long way in explaining why the controversy occurred and how it can be resolved.

The fact that cancer metastasis, therapeutic resistance, and immunosuppression are three complex and poorly understood processes, which often coexist clinically, is of particular note.^55,107,109^ Although metastasis rather than the primary tumor is the reason for ~90% of cancer-associated deaths,^129^ drug resistance must also be addressed. More recently, two seminal papers by Fischer et al.^118^ and Zheng et al.^145^ have highlighted an irreplaceable role of the EMT in cyclophosphamide and gemcitabine resistance of breast and pancreatic cancer cells, respectively, while challenging the conventional role of EMT in cancer metastasis.

Here, in this review, we focus on the mechanistic inter-relationships between the EMT programs—representative of cancer cell plasticity, and the resistance to cancer therapies, including targeted therapy, chemotherapy, immunotherapy, and radiotherapy.

### The relationship between EMT and drug resistance

It is commonly believed that re-activation of developmental programs is one of the principal mechanisms controlling many adult disease processes, including the EMT programs in drug resistance.^56,135,146^ To better understand the relationship between gene- and protein-expression profiles in tumor tissues of cancer patients and their corresponding clinical responses, multiple studies have been performed. These showed a positive correlation between the expression of mesenchymal-/stroma-related markers and therapeutic resistance, including for chemotherapy, targeted therapy, radiotherapy, and immunotherapy, although at times this has been controversial.^147–157^ For example, in the context of estrogen receptor (ER)-negative breast cancer, Farmer et al.^147^ have reported that upregulation of the genes within stromal metagene exhibits a significant predictive effect on the resistance to neoadjuvant chemotherapy with 5-fluorouracil, epirubicin, and cyclophosphamide. This signature seems to, at least in part, depend on the activation of EMT programs within carcinoma cells.^147^ Analogously, employing integrative analyses of gene expression and proteomic profiling, a robust 76-gene mesenchymal signature was derived and verified to have the potential to predict whether or not the resistance to EGFR-TKIs and phosphatidylinositol 3-kinase/protein kinase B (PI3K/Akt) inhibitors would be acquired in various NSCLC cell lines and clinical samples, highlighting the significant impact of different phenotypic (epithelial and mesenchymal) states on drug responsiveness.^148^ Furthermore, in melanoma, two markers (PTRF and IGFBP7) related to phenotype switching from melanocytic to mesenchymal state were shown to distinguish MAPK inhibitor-resistant cells from MAPK inhibitor-sensitive melanoma cells by proteomic screening.^149^ In short, considering all the above, the concept that EMT programs serve as a direct contributor to the acquisition of resistance to both cytotoxic and targeted therapeutic agents in a variety of cancer types is fairly convincing.

Over the years, the rapid development of immune checkpoint blockade (ICB) therapies (e.g., inhibitors of cytotoxic T lymphocyte-associated protein 4 [CTLA-4] and programmed cell death-1/programmed cell death-ligand 1 [PD-1/PD-L1]) has revolutionized the clinical treatment landscape in a wide range of advanced tumor types. Nevertheless, low response rates as well as ensuing immunotherapy resistance and delayed relapse represent a significant challenge in the field of cancer immunotherapy for the treatment of a variety of tumors, including lung adenocarcinoma,^158,159^ melanoma,^160,161^ PCa,^162^ and pancreatic cancers.^163^ However, the molecular mechanisms involved in immune escape remain elusive,^164^ and the influence of EMT programs on immunotherapies remains controversial. A number of studies have suggested a positive correlation between EMT-related signatures and T cell infiltration, leading to enhanced sensitivity to ICB.^150–154^ By contrast, others have indicated that tumors with EMT/stroma-related gene expression are closely connected with lower clinical responses and poorer progression-free and overall survival.^156,157^

#### Tumor microenvironment (TME) contributes to resistance via EMT

##### (1) TGF-β within TME

**Fig. 2:**
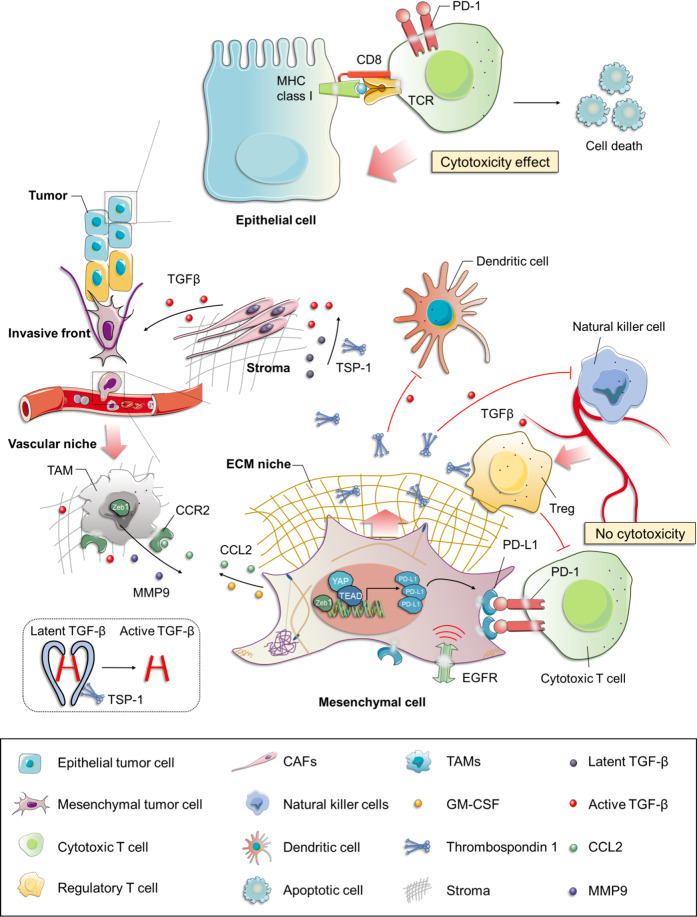
The tumor microenvironment (TME) contributes to resistance via EMT. Increased levels of TSP-1 (the activator of TGF-β1) secreted by mesenchymal tumor cells on the one hand facilitates the further activation of the TGF-β signaling pathway, contributing to a positive feedback effect on EMT; on the other hand, it promotes the generation of Foxp3^+^ Tregs from naive CD4^+^ CD25^−^ T cells that antagonizes the activity of cytotoxic T cells, together with the induction of impaired DCs and inhibition of NK cells within the TME, thus ultimately resulting in immunotherapy, and even chemotherapy resistance. Tumors arising from the mesenchymal cells express a higher level of PD-L1 and lower level of MHC-I, together with more Tregs within TME in comparison with those formed by the epithelial cells, supporting the immunosuppressive role of EMT programs, which at least in part contributes to the resistance to cancer therapies. The TAMs, known as the most plentiful immune-related stromal components in TME, have been shown to infiltrate mainly at the invasive fronts of tumors. CCL2, synthesized by cancer cells, triggers the recruitment of circulating monocytes with the expression of CCR2 into tumors with the subsequent acquisition of a TAM phenotype. ZEB1-expressing macrophages promote their own polarization toward a stronger protumor phenotype; and meanwhile, upregulate the expression of CCL2 and CD74 in cancer cells through an increased release of MMP9, resulting in a mesenchymal/stem-like state. This forms a CCR2-MMP9-CCL2^+^ feedback loop between TAMs and the cancer cells. TSP-1 thrombospondin-1, PD-L1 programmed cell death-ligand 1, Tregs regulatory T cells, DCs dendritic cells, MHC-I the class-I major histocompatibility complex, TAMs tumor-associated macrophages, CAFs cancer-associated fibroblasts

TGF-β, a well-established key promoter and sustainer of mesenchymal and/or CSC state,^165,166^ contributes to the induction and function of immunosuppressive regulatory T cells (Tregs)^167,168^ and inhibition of metabolic activity of natural killer (NK) cells,^169,170^ laying the foundation for molecular mechanisms underlying the significant role of EMT programs in antitumor immune response (Fig. 2). It is known that TGF-β can be activated by removing the N-terminal latency-associated peptide through serine proteases (plasmin^171^ and cathepsin D^172^) and matrix metalloproteinases (MMP9 and MMP14).^173^ Activated TGF-β can bind to a subset of integrins (including αvβ6,^174,175^ αvβ8,^173,176^ and αvβ1,^177^) or bind to the secreted and matricellular protein thrombospondin-1 (TSP-1, which is regarded as the first discovered activator of TGF-β1) under both physiological and pathological conditions in vivo.^178–182^ An increased level of TSP-1 secreted by mesenchymal cells, especially *Snail*-overexpressing cells, on the one hand facilitates the further activation of the TGF-β signaling pathway, thus contributing to a positive feedback effect on EMT. On the other hand, it promotes the continual generation of Foxp3^+^ Tregs from naive T cells, which antagonize the effects of cytotoxic T lymphocytes (CTLs), together with the induction of impaired dendritic cells (DCs) and inhibition of NK cells within the TME, thus ultimately resulting in resistance to immunotherapy, and even chemotherapy^183–186^ (Fig. 2).

To extend these observations further, epithelial and mesenchymal cell lines derived from the transgenic MMTV-PyMT breast cancer model have been established.^187^ Tumors arising from the more mesenchymal-like cell lines (with high levels of vimentin and EMT-TFs) exhibit the reduction of the class-I major histocompatibility complex together with more Tregs within TME in comparison with those formed by the more epithelial-like cell lines (with significant levels of E-cadherin and epithelial cell adhesion molecule).^188^ This is consistent with the immunosuppressive role of EMT programs described above. In addition, tumors arising from the more mesenchymal cell lines are frequently accompanied by an obvious infiltration of protumor and anti-inflammatory alternatively activated (M2) macrophages (CD206^+^ and Arginase1^+^), instead of antitumor and proinflammatory classically activated (M1) macrophages (inducible nitric oxide synthase, iNOS^+^ and IL-12^+^), which occur in tumors arising in the more epithelial cells.^188–190^ This mirrors the switchable potential between two different polarization states due to the high plasticity of macrophages depending on changes in the local TME—in this case, referring to the induction of EMT programs of cancer cells within the TME.^190–192^ While the binary system of polarization states seems an attractive hypothesis, accumulating evidence demonstrates that tumor-associated macrophages (TAMs) prefer to share a mixed phenotype and express both M1 (HLA-DR, interleukin-1β [IL-1β], or TNF-α) and M2 (CD163 and IL-10) markers rather than being considered as two cell types completely independent of each other.^193,194^ This may explain why TAMs exhibiting characteristics of either tumor killing (M1-like) or tumor-promoting (M2-like) macrophages can play an equally important role in the induction of EMT programs in PDAC.^195^

##### (2) The role of inflammation in EMT

The concept that there exists a causal link between chronic inflammation and the onset of cancer is well established and widely accepted owing to comprehensive biochemical and clinical evidence.^196–198^ Indeed, the effects exerted by inflammatory reactions on cancer is not limited to its initial stage, but can also be observed during its progression, including late-stage disease characterized by the acquisition of malignant behaviors, particularly those related to the induction of EMT.^198^ Before discussing the role of inflammation on EMT programs, it is beneficial to explore how inflammation is involved in other physiological and pathological processes, among which wound healing is highly representative.^199,200^ Of note, the implementation of wound healing or tissue regeneration, a highly conserved process that largely depends on an EMT-induced migratory phenotype of keratinocytes, tends to be a result of the combined effect of the inflammatory microenvironment and EMT, serving as a perfect paradigm for investigating the interactions between these two events. In this sense, when extending this paradigm to cancer progression, there is every reason to believe that such a crosstalk between inflammation and EMT can be replicated, hijacked, and extended by carcinoma cells, with EMT programs being exploited for the metastatic process. This echoes the previous notion that tumors have characteristics similar to long-term unhealing wounds.^201^ More importantly, cancer therapy, especially chemotherapy and radiotherapy, are both capable of stimulating inflammatory responses per se, providing the mechanistic foundation for the involvement of inflammation in therapeutic resistance, and even immunosuppressive TME.^202,203^ In this context, recent advances regarding the roles of inflammation and EMT programming, and their interactions in resistance will be summarized, with a particular focus on the functions of two sources of inflammatory cellular components, namely, macrophages and myeloid-derived suppressor cells (MDSCs).

- Recruitment of macrophages into TME and resistance. From the macroscopic viewpoint, apart from tissue-resident cells, there also exist a high proportion of multiple immune cells recruited to the TME, which have been termed as “partners in crime” in the “sanctuary of the devil.”^204,205^ Among these, the TAMs, known as the most plentiful immune-related stromal components in TME,^206^ might account, in part, for the poor prognosis of patients with different types of tumor, such as hepatocellular carcinoma (HCC),^207^ breast,^208–210^ gastric,^211–214^ lung,^215–217^ pancreatic,^218^ PCa,^219^ esophageal^220^ and thyroid cancers,^221^ and Hodgkin’s lymphoma.^222,223^ Therefore, not only the numerical superiority of infiltrating cells within the TME, but also the significant effect on patient prognosis indicates a pivotal role of TAMs in the crosstalk between cancer cells and TME.^224^ In general, the emergence of a TAM phenotype firstly requires for the recruitment of monocytes into TME triggered though various tumor cell-derived cytokines, chemokines, and growth factors in a collaborative way, which contain granulocyte–macrophage colony-stimulating factor (GM-CSF), chemokine (C-C motif) ligand 2 (CCL2), CSF-1, macrophage-stimulating protein, vascular endothelial growth factor*-*A, and TGF-β1, and so on. Subsequently, these recruited monocytes can further differentiate into M2 macrophages fueled by IL-4, IL-6, or IL-10.^190^
  - GM-CSF and IL-6. Similar to cancer-associated fibroblasts (CAFs), TAMs have also been shown to infiltrate mainly at the invasive fronts of tumors, the sites where cancer cells most frequently undergo EMT (Fig. 2). Such spatial overlap between host and tumor indicates that the signals resulting from bidirectional communications may be responsible for the commonly accepted spatial characteristics of EMT induction mentioned previously.^225–229^
    Based on that, Bonde et al.^230^ demonstrated a significant role for intratumoral TAMs in the activation of EMT programs in cancer cells through depletion of macrophages in F9-teratocarcinoma bearing mice, thus broadening and challenging the traditional view that TAMs-mediated EMT is confined to the invasive fronts. Furthermore, TGF-β derived from macrophages has been identified as the major cytokine controlling this highly context-dependent phenotype switching using a candidate-based screen. Supporting this notion, a systematic analysis of a large number of NSCLC tissue samples revealed that overexpression of EMT-associated markers in tumor cells was significantly and positively correlated with intratumoral CD68^+^ macrophage density and intraepithelial TGF-β levels, together with concomitant higher histologic grade and tumor heterogeneity, all of which contributed to drug resistance and patient relapse.^230^ In addition, by comparing the effects on human monocytes of coculturing with the medium derived from epithelial-like or mesenchymal-like cell lines, respectively, Su et al.^227,231^ reported that breast cancer cells with mesenchymal signature could activate macrophages to differentiate into the M2-macrophage phenotype, which was partially attributed to the secretion of GM-CSF from mesenchymal tumor cells (Fig. 2). This immunosuppressive phenotype, in turn, further strengthened EMT programming of tumor cells in various cancer types, including, but not limited to, breast,^227,232^ gallbladder,^233^ and pancreatic,^234^ as well as head and neck cancers,^235^ via releasing CCL18 from TAMs, forming a positive feedback loop both in vitro and in vivo^227,231^ (Fig. 2). A recent study that focused on investigating the role of oncoprotein MCT-1/MCTS1 (multiple copies in T cell malignancy 1) in triple-negative breast cancer (TNBC) identified significant enrichment of proinflammatory cytokines/chemokines, such as IL-6, GM-CSF, and monocyte chemotactic protein-1 (also known as CCL2) released from MCT-1-overexpressing cells compared to those observed in control cells.^236^ Among those cytokines, the relative abundance of IL-6 within TME has been demonstrated to promote the trans-polarization of infiltrating THP-1 monocytic cells into an immunosuppressive M2-like macrophages phenotype (CD163^+^ and CD206^+^);^237^ the activation of EMT processes, together with the maintenance of cancer stemness in TNBC, jointly result in the suppression of antitumor immune responses and tumor recurrences following therapy.^236,238,239^
  - CCL2. Aside from GM-CSF and IL-6, the chemokine CCL2, synthesized by cancer and stroma cells within the TME, triggers the recruitment of proinflammatory F4/80^low^ circulating monocytes with expression of CCR2 (the receptor for CCL2) into tumors with the subsequent acquisition of a TAM phenotype^240,241^ is emerging as a prominent regulator of cancer metastasis,^242^ especially metastatic growth,^243,244^ and angiogenesis.^245^ Interestingly, continuous recruitment and enhanced infiltration of macrophages resulting from upregulation of tumor cell-derived CCL2 is observed upon targeting androgen receptor (AR) with short interfering RNAs, which leads to the establishment of an immunosuppressive microenvironment, induction of EMT programming, and a growing population of stem/progenitor cells.^246,247^ These eventually result in the development of androgen deprivation therapy (ADT) resistance in PCa.^246,247^ Similar CCL2-mediated monocyte/macrophage trafficking was also observed in the inducible *Kras*^*G12D*^ *p53*-null (iKPC) PDAC mouse model, which largely depends on overexpression of *HDAC5.*^248^ Subsequently, TGFβ secreted by these recruited TAMs endows tumor cells with a mesenchymal-like phenotype enabling them to survive in case of oncogenic KRAS (KRAS*) extinction, indicating a significant role of the CCL2-TGFβ/EMT signaling pathway in the resistance to KRAS* targeting therapy.^248^ While a similar quasi-mesenchymal phenotype has already been validated by previous studies focused on the acquisition of resistance to KRAS* functional suppression both in KRAS*-driven PDAC and lung cancer,^249,250^ the results reported by Hou et al.^248^ extend the mechanisms involved in bypassing KRAS* addiction from the tumor cell per se (YAP1 activation) to host-tumor interactions in PDAC. Given the growing emphasis on the role of YAP1 or Hippo pathway in the TME,^251–253^ it seems worth exploring how CCL2 and YAP1 could be integrated together to promote EMT-related resistance.^254^
    Using subcutaneously implanted tumor models in mice with PDAC cell lines derived from spontaneous tumors of *Kras*^*LSL-G12D/+*^, *Trp53*^*LSL-R172H/+*^, and *Pdx1-Cre* (KPC), Kalbasi et al.^255^ demonstrated that the accumulation of tumor-derived inflammatory cytokines and chemokines, particularly a sharp increase of CCL2 compared to baseline in response to the stress of ablative radiotherapy, boosts the recruitment of Ly6C^+^CCR2^+^ monocytes/macrophages into the TME. Aided by this radiotherapy-induced macrophage trafficking, tumor cells acquire strengthened survival capacity and heightened intratumoral neovascularization, instead of T cell infiltration, ultimately giving rise to radiotherapy resistance in PDAC.^255^ Furthermore, the blockade of the CCL2–CCR2 axis by a neutralizing anti-CCL2 antibody significantly abrogates the recruitment and infiltration of inflammatory monocytes upon ablative radiotherapy, supporting a novel therapeutic role for targeting tumor-derived CCL2 against resistance to radiotherapy in PDAC.^255^ Because of the convincing evidence from many studies on various cancers, which highlight the contribution CCL expression has made to the activation of EMT programs,^256–259^ we put forward the hypothesis that the mechanisms underlying radiotherapy resistance involve the transition towards a mesenchymal phenotype in cancer cells with CCL2 expression. This hypothesis is in line with observed critical role of TAMs infiltration in EMT induction.
    More recently, based on a transgenic mice model of ovarian carcinoma, Cortés et al.^260^ demonstrated that the tumor-promoting functions of TAMs, as represented by chemotherapy resistance, requires full *Zeb1* expression by TAMs with the release of CCL2 by the cancer cells. It is generally known that expression of ZEB1 (the well-characterized key activator of EMT) by cancer cells endows them a more aggressive phenotype, including enhanced invasive capacities, therapeutic resistance, and stemness properties, resulting in poor clinical outcomes in a variety of human cancer types^261–263^ (Fig. 2). Rather than simply focusing on tumor cells, it is important to understand which stromal cell types also expresses ZEB1 and how these cells perform their functions within TME.^264^ Cortés et al. and other researchers showed that in the context of ovarian cancer, ZEB1-expressing macrophages promote their own polarization toward a stronger protumor phenotype (F4/80^low^, CCR2^+^),^265,266^ and meanwhile, upregulate the expression of CCL2 and CD74 in cancer cells through an increased release of MMP9, thus resulting in phenotype switching towards a mesenchymal/stem-like state of carcinoma cells^260^ (Fig. 2). This forms a CCR2-MMP9-CCL2^+^ feedback loop between TAMs and the cancer cells, which significantly contributes to resistance to chemotherapeutic drugs (e.g., cisplatin) due to the expression of ZEB1 by both cancer and stroma cells (TAMs) (Fig. 2).^260^ Targeting ZEB1 in cancer cells is currently being considered in clinical trials. However, the above data suggest that effective inhibition of tumor growth and improved response to chemotherapy would also require targeting of ZEB1 in TAMs.^260^
    Similar contributions by TAMs to the resistance of chemotherapeutics (gemcitabine and 5-fluorouracil) via EMT induction have been validated in other cancer types, including pancreatic and colorectal cancers.^267,268^ For this reason, strategies targeting TAM or involving EMT can be hijacked and exploited for therapeutic purposes by modulating TAM function, infiltration, or activation. Collectively, it follows that phenotypic and functional switching back and forth between epithelial and mesenchymal states plays a crucial role in the resistance to immunotherapy and the establishment of an immunosuppressive TME by its effect on multiple immune cell types, perhaps in a coordinated fashion.^189^
    Indeed, given the existing evidence detailed above, it is plausible that EMT-induced resistance to different therapeutic strategies, including targeted therapy, chemotherapy, immunotherapy, and radiotherapy, in certain cases seems to coexist simultaneously despite relying on different molecular mechanisms. This raises the possibility that these distinct biological processes interrelate closely with each other, similar to the role played by TAMs, based on the inflammatory microenvironment.
- Interactions between MDSCs and EMT induce resistance. Together with the positive feedback role that TAMs, growth factors (i.e., TGF-β) and chemokines (i.e., CCL18) play in the EMT programming of carcinoma cells, the recruitment of other inflammatory cells within TME and a surge of tumor-promoting soluble factors associated with inflammation, as well as the activation of key inflammatory signaling pathways, also promote the malignant behaviors of multiple cancers, especially those in relation to the EMT induction—including resistance and metastasis.^55,269,270^ MDSCs, a heterogeneous, immunosuppressive population of immature myeloid cells, which tends to be accumulated within TME under chronic inflammation^271,272^ can be used as an exemplar. These heterogeneous MDSCs, characterized by the multiplicity and complexity of their phenotypic markers, have been classified into two main categories: monocytic MDSC (mMDSC) and polymorphonuclear or granulocytic MDSC (PMN-/Gr-MDSC).^273^ Using RETAAD (the activated *RET*) transgenic mouse model of melanoma, a comparative analysis of immune infiltrates from primary and metastatic sites noted that CD11b^+^Gr1^hi^F4/80^−^ PMN-MDSCs could be selectively recruited to and infiltrate in the primary tumor mass, where inflammatory cells are relatively plentiful, by CXCR2-CXCR2 ligand (i.e., CXCL5) interactions. This contributes to the induction of EMT and its associated tumor dissemination and therapeutic resistance.^274^ Additionally, in a lethal PCa model triggered by deletion of *Pten* and *Smad4*,^275^ a similar communication between cancer and tumor-associated inflammatory cell, that is, an elevated recruitment of CXCR2-expressing MDSCs attracted by upregulated expression of CXCL5 in the carcinoma cells, has been identified. This largely depends on Hippo-YAP signaling in a non-cell-autonomous manner.^276^ On the basis of such a dependency, coupled with the well-recognized cell-autonomous role of YAP1 and the involvement of CXCL5 in EMT,^250,274,277^ it is reasonable to hypothesize that a subpopulation of cancer cells characterized by a mesenchymal signature may be localized at invasive fronts, which would facilitate the establishment of an immunosuppressive TME through selective secretion of chemoattractants like CXCL5, thus resulting in resistance and metastasis.
  In addition to the similarities detailed above, different and sometimes opposing effects of PMN-MDSCs involvement in EMT programming in multiple cancer types are worth to be acknowledged, thereby allowing the heterogeneity of phenotypes (cell-surface markers) to connect with that of functions. For example, based on syngeneic mouse models of mammary carcinoma, Ouzounova et al.^278^ demonstrated that a preferential, regional recruitment of PMN-MDSC to the lung facilitated the establishment of a premetastatic, inflammatory environment, which could induce, to some extent at least, DTCs to regain epithelial characteristics, particularly the fast-growing phenotype, by activating MET programs, ultimately resulting in colonization and overt pulmonary metastases in vivo.^279^ In contrast, the model also showed that mMDSCs infiltrated and gathered at the invasive fronts of the primary tumor, tending to play a role in the process of tumor dissemination by inducing a motile, drug-refractory, mesenchymal-like phenotype.^278^ Such an enhanced migration of mMDSCs to the primary tumor has been further validated by positron emission tomography imaging in a PyMT breast cancer model,^279^ and also observed in mice bearing platinum-resistant bladder tumors,^280^ implying a potential role for anti-inflammatory therapy (in this case, MDSC-targeted therapy) in increasing the susceptibility of cancer cells to antitumor drugs.
  Supporting this notion, the significance and feasibility of the above therapeutic strategy has been enhanced due to the robust curative effects in a chimeric mouse model of metastatic castration-resistant PCa, which were achieved by a combination of immune checkpoint inhibitors with anti-MDSC agents (e.g., cabozantinib [Cabo] and BEZ235 [BEZ]).^162^ Mechanistically, the success of such synergistic responses largely depends on the Cabo- and/or BEZ-induced diminishment of intratumoral Gr-MDSCs (CD11b^+^Gr1^+^Ly6G^+^Ly6C^low^) and reduction in secretion of MDSC-promoting cytokines (e.g., CCL5, CCL12) from carcinoma cells without impairing the function of CTLs.^162^ Further studies indicated that these recruited Gr-MDSCs, also known as PMN-MDSCs, were predominantly enriched in both human castration-resistant prostate cancer (CRPC) biopsies and castrated mice tumors, which could in turn release IL-23 to promote acquired resistance to androgen-deprived therapies by upregulating AR signaling in PCa cells.^281^ IL-23 is a heterodimeric and immunomodulatory cytokine that, when activated inappropriately in esophageal cancer (e.g., secreted by MDSCs within the TME) can result in protumor inflammatory responses and immune escape,^282^ during which the EMT programming is involved.^283^ These observations provide direct evidence to support the view that MDSC-mediated therapeutic resistance seems to be a consequence of the synergistic action of tumor and recruited host cells, including the induction of EMT in carcinoma cells and MDSC-induced immunosuppressive TME through a bidirectional, chemokine–cytokine crosstalk mechanism. However, from a more macroscopic level, in the context of drug resistance, the elementary question about the causal relationship between EMT and MDSCs, namely whether the mesenchymal signature of cancer cells can cause the recruitment of MDSCs by releasing chemoattractant chemokines, or can be caused by these recruited MDSCs through secretion of cytokines, remains unanswered. Further studies, centered on the functional and mechanistic links between EMT and MDSCs, as well as their relevance to therapeutic response, are therefore needed.

##### (3) Hypoxia signaling in driving EMT and resistance

The contribution made by hypoxia to cancer progression and therapeutic resistance has long been observed in a wide spectrum of cancers, since it was demonstrated by Wenger and colleagues that inactivation of hypoxia-inducible factor (HIF) could sensitize carcinoma cells to chemotherapeutic agents, including carboplatin and etoposide.^284,285^ The mechanistic basis for the effects of hypoxia on drug resistance is complex and varies from cancer to cancer. In general, hypoxia can impede drug sensitivity by manipulating drug efflux, cell proliferation and survival signaling pathways, DNA damage repair, metabolic reprogramming, tumor vascularization, stemness maintenance, and modification of stromal cells.^286–289^ Although the mechanism underlying hypoxia-mediated drug tolerance is not fully understood, the effect of EMT has attracted major attention.

In several incidences of EMT, including cancer and fibrosis, hypoxia is experienced as a dynamic stimulus in the local microenvironment under ischemic conditions.^290^ Therefore, it is reasonable to infer the existence of a crosstalk between hypoxia and EMT. The influences of low oxygen on cancer cells are orchestrated by HIF.^291^ HIF-1α is a TF, which can be degraded by prolyl hydroxylases, such as PHD2, under normoxic conditions.^292^ Notably, lack of oxygen can inactivate PHD, leading to the accumulation and subsequent activation of HIF-1α. Activated HIF-1α can directly bind to the hypoxia-responsive element of the promoter of several EMT-associated genes, such as *TWIST1* and *TGF-β*, to stimulate the induction of EMT.^293^ Furthermore, HIF-1α can also promote EMT through mediating PI3K/Akt, Wnt, and Notch signaling pathways. For instance, HIF-1α cooperates with N1ICD as a transcriptional complex to be recruited to the *Snail* promoter, thus promoting SNAIL expression. In addition, Notch can potentiate the recruitment of HIF-1α to the lysyl oxidase (LOX) promoter and enhance the expression of LOX, which can further stabilize the Snail protein and induce the EMT process.^294^ In addition, hypoxia can also induce EMT by regulating the communication of exosomes derived from bone marrow-derived mesenchymal stem cells (MSCs) and cancer cells.^295^ Indeed, hypoxia confers cancer cells with cues for preserving a stem-like phenotype and bridges the linkage between EMT and drug resistance.^290^ For example, hypoxia in the central region of HCC considerably decreases the drug sensitivity of tumor cells through inducing EMT programs. Salidroside can promote the degradation of HIF-1α, thus inhibiting the EMT of HCC cells, leading to enhanced antitumor efficacy of platinum drugs.^296^ Furthermore, hypoxia can activate EMT by activating the nuclear factor-κB (NF-κB) pathway, evidenced by the observed EMT-like morphology and EMT protein markers in hypoxic lung cancer cells. 20(*R*)-Ginsenoside (Rg3), known as the ginseng extract, can increase the sensitivity to cisplatin in hypoxic lung cancer cells by inhibiting EMT.^297^

##### (4) EMT-induced immunosuppressive TME in drug resistance

In the context of the TME, contributions made by EMT programs to treatment resistance are reflected in two major drivers: one is the secretion of cytokines and/or chemokines derived from non-tumor cells that triggers the phenotype switching of epithelial cancer cells towards a mesenchymal state, directly resulting in drug resistance via EMT itself; occurring in parallel are alteration in the distribution and function of multiple tissue-resident cells and/or recruited immune cells within TME, which is a consequence of the EMT induction of cancer cells either directly or indirectly. This creates an immunosuppressive microenvironment upon drug exposure, ultimately giving rise to immune escape and therapy resistance.^298–300^ Further studies are required to address the coexistence and dependence of these two aspects, and the underlying interlinkage mechanisms by which EMT programs and immunosuppression function together to evade the lethal effect of antitumor drugs.

- EMT and PD-L1 expression. The positive correlation between EMT programs and the expression of PD-L1 (also called as B7-H1 or CD274), a ligand binding to the immune receptor PD-1 (also known as PDCD1), widely occurs in healthy tissue cells, antigen-presenting cells and a variety of tumor cells for escaping antitumor immune responses. It serves as a “bridge” to connect EMT programs and immunosuppression.^153,301,302^ For example, based on the EMT signature in lung cancer previously mentioned,^148^ an analysis of patient-derived, pan-cancer EMT signature reveals that tumors of mesenchymal status exhibit significant enrichment of multiple immune checkpoints, especially PD-L1, which may act as novel biological targets or therapeutic vulnerabilities in mesenchymal-like tumors.^150^ Even before this was realized, studies had built a large body of credible evidence for the contributions made by PD-1/PD-L1 inhibitory pathway to immune escape. This was supported by the following facts: inhibition of CD8^+^ CTL proliferation and function, and enhanced production of Foxp3^+^ Tregs from CD4^+^ T cells, resulting in peripheral immune tolerance^303–306^ of patients in several types of carcinomas, including NSCLC,^307,308^ lung squamous cell carcinoma,^303^ liver cancer,^309^ and myeloproliferative neoplasms.^310^ This functional connectivity suggests that the expression of PD-L1 in cancer cells, in some sense at least, may play a role as an indicator since the levels of immunosuppression rise during cancer progression. This has led to intensive discussions about the relationship between EMT programs and immunosuppression, with a particular emphasis on PD-L1.
  Subsequently, following a succession of recent studies, a similar positive relationship between EMT programs and PD-L1 expression has been further validated in multiple cancer types, including lung,^153,301,302^ breast,^188,311^ and head and neck cancers.^312^ Furthermore, this relationship has the potential to perform in a bidirectional regulating and controlling mode. In general terms, the induction of EMT can cause significant upregulation of PD-L1. In turn, several studies have shown that the activation of PD-L1 signaling is also crucial for maintaining the characteristic manifestation of malignant tumors with aggressive clinical features, in particular EMT programming,^311,313,314^ immune escape,^307,308^ and stem cell properties.^315,316^ Consequently, higher expression of PD-L1 has been connected to worse prognoses in multiple types of carcinomas, including esophageal cancer,^317^ renal cell carcinoma,^318^ gastric carcinoma,^319^ ovarian cancer,^320^ and melanoma.^321^
  Reinforcing this concept, a raft of studies have suggested that the miR-200/ZEB1 axis, a well-understood double-negative feedback loop that governs the reversible phenotypic transformation of cancer cells between epithelial and mesenchymal states,^322–325^ participates in multiple processes of auxiliary cellular functions associated with cancer progression, including immune escape,^154,260,326,327^ drug resistance,^260,328,329^ stem cell properties,^330^ and endothelial trans-differentiation.^331,332^ It is especially interesting that under most conditions, the target genes for these functionally overlapping biologic processes are more likely to function in a non-overlapping mode, which means the EMT-related miR-200/ZEB1 axis is endowed with an incredible potential for regulating pleiotropic downstream targets specialized in handling various cellular functions.^154,333–335^ With increasing in-depth studies, the list of target genes is constantly being expanded. In line with this, *PD-L1* has been identified as a direct downstream target of the miR-200/ZEB1 axis in NSCLC cells by Chen et al.,^154^ resulting in a diminished antitumorigenic immune response due to CD8^+^ T cell (PD-1^+^ TIM-3^+^) exhaustion and a reduced number of CD8^+^ TILs.^326^ Similar mechanisms have been further validated by various studies on breast cancer and NSCLC.^327,336^ In short, these observations provide direct experimental evidence that EMT induction and PD-L1-mediated immunosuppressive TME are closely associated via miR-200/ZEB.^154^ In addition, a retrospective study on colon cancer at the histological level has shown that the regulation of miR-200/ZEB axis, manifesting itself as upregulated expression of ZEB and downregulated expression of miR-200, occurs preferentially in regions of tumor budding at the invasive front where EMT is frequently accompanied by significantly elevated expression of PD-L1, rather than at the tumor center.^337,338^ This spatial overlap can also be interpreted as a consequence of the close interactions between EMT programming and PD-L1 expression in cancer cells. From a macroscopic perspective, it may explain the observations that carcinoma cells at the invasive front, supposedly more vulnerable to autoimmune attack because of direct exposure to immunocytes, exhibit improved survival and a capability of avoiding host immune surveillance.
  These findings have gained further support from another study, which indicated the activation of *PD-L1* transcription can be driven by Mucin 1-C (MUC1-C)-induced formation of transcription initiation complexes with NF-κB p65 on the *PD-L1* promoter.^339^ These MUC1-C–NF-κB p65 complexes are also recruited to occupy the promoter of other NF-κB target genes, comprising *MUC1-C* and *ZEB1*, among which the former results in an auto-inductive circuit, while the latter leads to the repression of miR-200c expression; in turn, the transcriptional activation of *ZEB1* in NSCLC cells triggers EMT programming through the negative feedback regulation of the ZEB1/miR-200c axis.^65,339–342^ Echoing the previous finding that *PD-L1* acts as a target of miR-200, it seems quite logical and reasonable to speculate that the MUC1-C functioning in conjunction with NF-κB p65 drives the induction of EMT and upregulates *PD-L1* gene expression at both the transcriptional and post-transcriptional levels via a ZEB1/miR-200-dependent mechanism. This raises the possibility that MUC1-C may act as an upstream regulator coordinating these two responses.^154,339^ As a consequence of this coordination, researchers have hypothesized that patients with mesenchymal tumors are more likely to benefit from immunotherapy, particularly for anti-PD-L1 or PD-1-neutralizing antibodies against PD-L1 overexpression.^122^ The possible role of PD-L1 as a prognostic factor in patients undergoing PD-1/PD-L1 inhibitor treatment is still under investigation and needs further studies and testing in clinical practice. As far as the current research findings are concerned, it may be concluded that patients whose tumors show high expression of PD-L1 (mesenchymal signature) may experience improved clinical outcomes with anti-PD-1/PD-L1 treatment. However, this is not always the case. Some patients with PD-L1-negative cancer (epithelial signature), across a wide variety of human cancer types, also show robust responses to PD-1/PD-L1 antibodies (e.g., nivolumab), suggesting that PD-L1 overexpression in tumor tissues is neither a sufficient nor necessary condition for guaranteeing improved clinical benefits (survival time) for patients.^343–349^
  A change of approach to predict patient response to immunotherapy may therefore be required. Emerging evidence shows that exosomes secreted by tumor cells have bioactive PD-L1 on their surface, which can suppress the immune response.^350–354^ Based on the clinical data of patients with melanoma or NSCLC,^351,355^ exosome-derived PD-L1 in response to treatment with anti-PD-1/PD-L1 antibodies (e.g., nivolumab and pembrolizumab) may have the potential to predict the clinical outcomes of anti-PD-1 therapy, or even to become a novel therapeutic target, in spite of the ambiguity of the relationship between EMT and PD-L1 levels within exosomes.
  Besides playing an essential role in immunosuppression and a possible role in resistance to immunotherapy as discussed previously,^356^ it is interesting to note that the “bidirectional regulation between EMT and PD-L1” is also involved in resistance to targeted therapy^357^ as well as chemotherapy, linking it to functions beyond its immunoregulation activities. Researchers have demonstrated that upregulation of PD-L1 mediated by YAP at the transcript level leads to the acquired resistance to EGFR-TKIs (e.g., gefitinib) in NSCLC, a process that largely depends on the induction of EMT.^357–360^ Similar findings have also emerged for malignant pleural mesothelioma, an extremely aggressive cancer originating from membrane covering the lungs and the inner side of the ribs.^361–363^ Besides making important contributions for the acquired resistance to gefitinib (a first-generation TKI), elevated PD-L1 expression also exerts a positive effect on the acquisition of intrinsic resistance to EGFR-TKIs in EGFR*-*mutant NSCLC by inducing an EMT phenotype, which might rely, at least partially, on the activation of the TGF-β/Smad canonical signaling pathway.^364–367^ These observations, together with the aforementioned bidirectional regulation between EMT and PD-L1, suggest that phenotypic switching between epithelial and mesenchymal states in carcinoma cells is usually accompanied by a dynamic transcriptional fluctuation of PD-L1 at the single-cell level.^16^ This enables PD-L1-expressing cells to survive following exposure to molecularly targeted agents. More importantly, activation of the EGFR pathway in turn induces upregulation of PD-L1 in EGFR-mutant NSCLC cells by alternative mechanisms, including the IL-6/Janus kinase (JAK)/signal transducer and activator of transcription 3 (STAT3)^368^ and ERK1/2/c-Jun^307^ signal transduction pathways. Similar instances of EGFR-driven PD-L1 expression have been observed in malignant melanoma cells (A875 and A375)^321^ as well as salivary adenoid cystic carcinoma (SACC) cells (SACC-83 and HN13) that underwent EMT,^369^ although the underlying molecular mechanisms vary from cancer to cancer.^308,321,368,369^ In addition to promoting PD-L1 expression, through a combined investigation of human glioblastoma (GBM) specimens and cell lines, EGFR signaling has also been characterized by its ability to maintain the stability of PD-L1 protein in a COP9 signalosome 6-dependent manner in which EMT programs are presumably involved.^370–372^ Indeed, the knowledge that activated EGFR plays an important role in adjusting PD-L1, either by promoting the expression or maintaining the stability of PD‐L1 protein, suggests that PD-L1 is less like a “bystander,” but rather a key participant in promoting biological functions of EGFR signaling. This would explain why the upregulated PD-L1 has been shown to more frequently occur in EGFR-TKI-resistant carcinoma cells.^365,367,373,374^ From the above studies, it can be reasonably assumed that the two therapies—targeted therapy (EGFR-TKIs, BRAF inhibitors, etc.) and immunotherapy (PD-1/PD-L1 or CTLA-4 blockade), when combined, could be a potential approach for improving outcomes for patients with resistance to either therapy alone. However, investigations on the clinical feasibility of this combined strategy remain at the initial stage due to the high frequency of adverse reactions, represented by immune-related adverse events and even interstitial pneumonitis in NSCLC patients,^375–378^ as well as hepatotoxicity in patients with melanoma.^379^
  Given the +nd on the mutual interplay between EMT programs and PD-L1 in certain types of human cancer.^357–363^ Such a dependency, consistent with the observations on resistance to targeted therapy, has been further confirmed from the acquisition of resistance to various cytotoxic chemotherapies. For instance, in HCC cells, transcriptional upregulation of PD-L1 mediated by Y-box binding protein-1, a multifunctional transcription/translation factor shown to be important in the regulation of multidrug resistance gene (*MDR1*) and EMT inducers (*Zeb1, Snail1*, *Twist*, etc.) by being recruited and binding to their promoter regions or mRNAs,^380–384^ results in an immunosuppressive TME (decreased CD8^+^ T cells and increased Tregs) and *MDR1* overexpression, as well as simultaneous EMT induction.^385^ Because of the extensive functional links among these biological processes, it is most likely that the acquisition of resistance in HCC cells to multiple chemotherapeutic drugs—particularly doxorubicin (DOX), is a consequence of joint, but not isolated, effects of the three aspects mentioned above.^385^ These results are consistent with previous research reported by Li et al.,^386^ using a comparative analysis between chemo-resistant and chemo-sensitive breast cancer cell lines and patient samples, demonstrated that high levels of PD-L1, together with low levels of miR-3609, are crucial for a heightened resistance to DOX and poor patient prognosis. In addition, another study that used the CRISPR/Cas9 system to probe the role of PD-L1 in the acquisition of chemoresistance showed that knockout of PD-L1 in osteosarcoma cells can significantly lower their resistance to DOX, and paclitaxel,^387^ further supporting the aforementioned studies.^385,386^ In addition to being involved in DOX resistance, the contributions to chemotherapy resistance made by reciprocal and complementary interactions between EMT and PD-L1 have been further validated in cisplatin (DDP) resistance of NSCLC cells. A possible mechanism in this study was attributed to the activation of the JAK/STAT3 pathway in an ataxia telangiectasia mutated-dependent manner,^388^ largely in agreement with the previous reports.^368,389^ Collectively, these observations provide strong evidence that the tight interactions between EMT programming and PD-L1 expression contribute critically to the development of resistance to either chemotherapeutics or targeted drugs, with or without the involvement of EMT/PD-L1-related immune escape.
- EMT and other immune checkpoints. As discussed above, immunotherapy based on immune checkpoint blockers has indeed revolutionized the therapeutic strategies for cancer; however, this treatment is still in its infancy owing to a lack of understanding regarding the interactions between tumor and host cells within TME, especially immune cells, during immunotherapy. Among the complicated dynamic, interactions between cancer cells and immune modulators, particular attention here should be paid to the induction of EMT because of its strong relevance to the expression of immune checkpoints, as well as their regulators or ligands like the aforementioned PD-L1. This can provide insight into the potential value of hijacking EMT signatures for therapeutic purposes, such as for improving response to immunotherapy.^390,391^ Supporting this concept, it has been widely reported that mesenchymal tumors are more refractory to immunotherapy, indicating that targeting the EMT process in combination with immunotherapy may hold great potential for improving the clinical outcome of immunotherapeutic strategies.^52^ In parallel, another study showed that Snail-expressing breast cancer cells exhibited reduced susceptibility to CTLs-mediated lysis.^392^
  Indeed, current observations suggest that the biological effects exerted by EMT on different immune checkpoints prefer to coordinate with each other, jointly promoting the establishment of an immunosuppressive TME.^391^ This EMT-mediated immune escape has been regarded as a major driver of resistance to multiple cancer therapies, including chemotherapy, targeted therapy, and immunotherapy. From this perspective, in order to overcome intratumoral immunosuppression, developing an in-depth knowledge of exactly how other immune checkpoints (i.e., excluding PD-L1) are regulated by EMT is of great importance and urgently required. To address this, Noman et al.^393^ have found that EMT-associated TFs (i.e., ZEB1 and Snail) play essential roles in regulating the expression of CD47 in human mammary cancer cells. Similar to the influence on PD-L1, Snail1/Zeb1 can directly bind to the E-box motif of the *CD47* promoter and thus induce their expression, which is closely linked to poor outcomes for patients with TNBC.^327,393,394^ In addition to CD47, the expression of CTLA-4, B and T lymphocyte attenuator, B7-H3 (CD276) and T cell immunoglobulin, and mucin protein-3 (TIM-3), along with the ratio of tumor immunosuppressive CD4^+^Foxp3^+^ Treg cells, are positively related to EMT phenotype in lung adenocarcinomas.^153^ In addition, a highly positive correlation has also been identified between EMT and newly emerging immune checkpoint-associated genes, including *CD276*, *TGFB1*, and *OX40* in kidney cancer.^395,396^ However, the molecular basis of EMT-mediated dysregulation of these checkpoint genes warrants further elucidation to provide insights for the use of EMT signatures as predictive biomarkers for ICB compounds and other immunotherapies in a broad range of cancers. Despite the prominent links between EMT and immunosuppression, the mechanism underlying EMT-mediated dysregulated immune checkpoint-associated genes still remains largely unknown and further investigations are urgently needed.

#### The role of EMT in resistance to EGFR-TKIs

In the previous section, the means by which PD-L1 expression on EMT programs contributes to the acquisition of resistance during EGFR-TKIs treatment has been extensively reviewed. We now explore the mechanics of EMT induction during the development of drug resistance for three generations of EGFR-TKIs, focusing on the tumor cells per se.

**Fig. 3:**
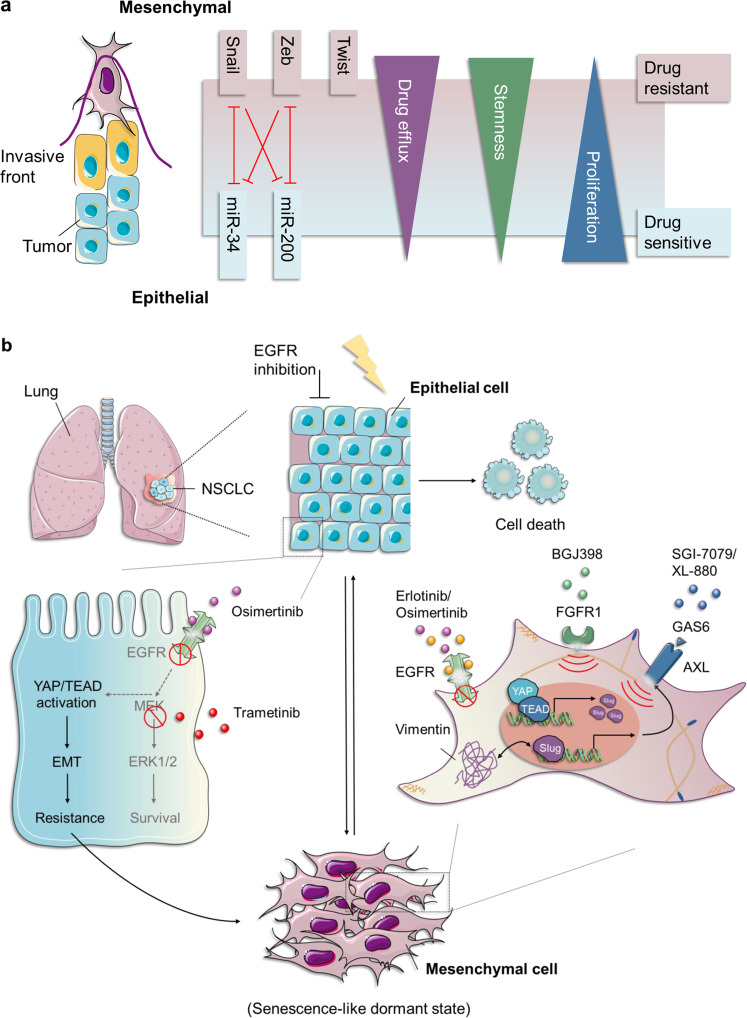
The role of EMT in EGFR-TKI resistance. **a** Cancer cells undergoing EMT are characterized by morphotypic switching from “cobblestone-like” shapes to “fibroblast-like” forms. This process can be achieved via several EMT-TFs (Snail, Zeb, and Twist) and miRNAs in response to paracrine and autocrine stimuli, endowing cancer cell with a more aggressive phenotype, including enhanced invasive capacity, therapeutic resistance (enhanced drug efflux and slow cell proliferation), and stemness properties. **b** In *EGFR*-mutant NSCLC, upregulation of TEAD-mediated YAP promotes the transcription of *Slug*, which further induces the upregulation of AXL in NSCLC cells. AXL signaling, whose activation relies on interactions with its specific ligand GAS6, promotes EMT that drives Slug-overexpressing mesenchymal cells to acquire resistance with erlotinib. In addition, the mesenchymal cells display enhanced resistance to EGF816 accompanied by a significant activation of the FGFR1 pathway, implicating the potential of FGFR1 as a drug target for evading resistance to EGF816. A subpopulation of cancer cells can enter a senescence-like state to escape cell death upon administration of EGFRi (osimertinib) in combination with MEKi (tretinamib), resulting in resistance. This change is characterized by YAP/TEAD-mediated activation of EMT programs. The therapeutic strategy of pharmacologically cotargeting YAP/TEAD (by MYF-01-37) and EGFR/MEK leads to synthetic lethality. AXL anexelekto, GAS6 growth arrest-specific protein 6, SGI-7079/XL-880 AXL inhibitor, EGF816 the third-generation EGFR-TKIs, FGFR1 fibroblast growth factor receptor 1, BGJ398: FGFR inhibitor

Initially, based on extensive experimental evidence from studies on *EGFR*-mutant NSCLCs, the features of EMT represented by the downregulation of E-cadherin, together with overexpression of vimentin and/or EMT-TFs Snail1/2, were observed in the course of overriding cytostatic effects of EGFR inhibition first-/second-generation EGFR-TKIs (such as gefitinib, erlotinib, and afatinib) or antagonistic monoclonal antibodies, prior to the development of genetic resistance mechanisms.^47–52^ The Hippo pathway, involved in tumor suppressor signaling, plays a critical role in the regulation of development, cell fate (e.g., cell senescence, proliferation, and apoptosis), and organ size, mainly by repressing the oncogenic TFs YAP and its paralog transcription co-activator with PDZ binding motif (TAZ).^397–401^ Previous studies have delineated the role of YAP/TAZ in the acquisition of drug resistance and the promotion of EMT programming in multiple cancers, including PDAC,^249^ melanoma,^402,403^ neuroblastomas,^404^ PCa,^405^ HCC,^406^ and lung cancer (ALK-rearranged,^407^ KRAS-mutant,^250^ and EGFR-mutant NSCLC.^360^) In *EGFR*-mutant NSCLC, more specifically, upregulation of TEAD-mediated YAP promotes the transcription of the EMT‑TF *Snail2* (*Slug*), but not *Snail1*, *Twist*, or *Zeb1*, which further induces the upregulation of the membrane-protein anexelekto (AXL) receptor tyrosine kinase in NSCLC cells. In this case at least, AXL acts as a downstream target of YAP^148,360,408–412^ (Fig. 3b). AXL signaling, whose activation relies on interactions with its specific ligand—growth arrest-specific protein 6 (GAS6), has been proven to be inextricably linked to the acquisition of the classic EMT-related signature,^413–415^ notably vimentin^411^ and E-cadherin^416,417^ (Fig. 3b). In this context, activated AXL directly drives Slug-overexpressing mesenchymal cells to acquire resistance with erlotinib in the absence of secondary mutations, such as the EGFR p.Thr790Met (T790M) alteration or MET activation^408,418,419^ (Fig. 3b). On the other hand, Slug overexpression can also antagonize p53-mediated apoptosis by repressing the transcription of proapoptotic effector PUMA, further promoting mesenchymal cell stemness and resistance to both targeted therapy (e.g., erlotinib) and chemotherapy (e.g., cisplatin).^420–422^ As expected, using the EGFR inhibitor (EGFRi) erlotinib in combination with the AXL inhibitor (SGI-7079/XL-880) or YAP1 inhibitor verteporfin can significantly diminish the expression of EMT-related markers and successfully restore the sensitivity to erlotinib of NSCLC cells with a mesenchymal signature, both in drug-resistant cell lines as well as in mouse xenograft models.^148,408–410^

Taken together, these results reveal that blockade of the GAS6/AXL pathway is sufficient to increase erlotinib susceptibility by reversing the EMT process, suggesting potential evidence for the treatment of advanced NSCLCs. Accordingly, the development of AXL inhibitors has already gained substantial interest from both academia and pharmaceutical companies. BGB324, known as a first-in-class AXL-selective small-molecule inhibitor, was developed by BergenBio and entered phase I clinical trials in 2013.^423^ Subsequently, a phase I/II clinical and pharmacokinetic study of BGB324 confirmed its safety and efficacy in patients with advanced NSCLC, a number of whom had sustained responses for at least 6 months without disease progression.^424^ A review of current strategies that target AXL, including application of small-molecule inhibitors, anti-AXL monoclonal antibody (20G7-D9),^425^ and high-affinity AXL decoy-receptor (MYD1-72),^426,427^ shows the multitarget AXL inhibitors (such as SGI-7079 and sunitinib) as combination partners have achieved the best curative effect with respect to drug resistance according to available preclinical and clinical evidence.^148,415,428,429^

In addition, treatment with the third-generation, irreversible EGFR-TKIs osimertinib (AZD9291), which has successfully doubled the median progression-free survival compared to that of the first-generation drugs, has been approved for the treatment of late malignant EGFR-mutant NSCLC and recommended as the preferred first-line therapy for those patients.^430,431^ Importantly, osimertinib, designed to target activated *EGFR*- and T790M-resistant mutations instead of wild-type *EGFR*, also remains the preferred second-line therapy for patients with advanced first-/second-generation TKI-resistant NSCLC.^432–434^ However, despite an initial obvious curative effect initially, patients usually suffer tumor recurrences within 1 to 2 years treatment with osimertinib due to acquired resistance.^357^ Prior studies based on clinical or preclinical models have revealed several mechanisms of acquired resistance to osimertinib, such as the secondary mutation (EGFR C797S),^7,435,436^ bypass pathway activation (*MET* and *HER2* amplifications),^437,438^ and an increased dependence on RAS signaling.^439^ These resistance mechanisms are mostly caused by genetic alterations, but non-genetic resistance mechanisms also exist, such as EMT induction, which is also necessary and sufficient to develop acquired resistance.^440^ Interestingly, consistent with the above mechanism, the GAS6/AXL axis plays a vital role both in the development of de novo resistance to osimertinib and also in the initiation and maintenance of a “persistent” state from EMT induction with or without re-activation of HER3 and EGFR.^440,441^ Therefore, the combination of the AXL inhibitor (Cabo or NPS1034) or the AXL degrader (Yuanhuadine, YD) with osimertinib is expected to be an effective therapeutic strategy for delaying or overcoming resistance in EGFR-mutant NSCLC, as seen in both in vitro and in viv*o* experimental models^440–443^ (Fig. 3b). Moreover, by the establishment of gefitinib- and osimertinib (AZD9291)-resistant NSCLC cell lines,^7,444^ representing first- or third-generation EGFR-TKIs, respectively, a recent study has demonstrated that the EMT induction tends to be a general phenomenon during the formation of acquired resistance in both situations when compared to parental cell lines.^445^ Although the underlying mechanisms of the above two cases are different, one depending on the activation of Src/Hakai, while the other depends on the upregulation of Zeb1, the mesenchymal, drug-refractory phenotype of gefitinib- and osimertinib-resistant NSCLC cells can be reversed via the simultaneous double inhibition of histone deacetylase (HDAC) and 3-hydroxy-3-methylglutaryl coenzyme A reductase using JMF3086.^445^ Based on these observations, Raoof et al.^446^ used mesenchymal cell lines derived from tissue samples of NSCLC patients suffering from TKI-resistant tumor, which simulated EMT-like phenotype switching. Using whole-genome screening, these mesenchymal cells showed increased resistance to the third-generation EGFR-TKIs (EGF816) in vitro accompanied by a significant activation of the fibroblast growth factor receptor 1 (FGFR1) pathway, implicating the potential of FGFR1 as a druggable target for evading resistance to EGF816^446^ (Fig. 3b). As expected, the novel use of EGF816 in combination with FGFR inhibitor BGJ398 can significantly inhibit the survival of mesenchymal cells and the development of full resistance in EGFR-mutant NSCLC^446^ (Fig. 3b). Collectively, from the aforementioned observations, as well as previous findings that the tumors undergo drug-induced phenotype switching while maintaining their primary mutations in EGFR, one can conclude that tumor cell plasticity-induced EMT is essential for acquired resistance upon multiple TKIs treatment, during which there are no genetic alterations.

Prevention of EGFR pathway re-activation by pharmacologically inhibiting downstream pathway components, such as RAF, MEK, or ERK, is another common strategy that has been implemented to delay resistance.^447,448^ Studies have indicated that, in comparison to treatment with the single-agent EGFRi (WZ4002), combination therapy with both WZ4002 and an MEK inhibitor (MEKi) (tretinamib/selumetinib) can significantly slow down resistance in *EGFR*-mutant NSCLC.^447^ However, even with this combined therapy, acquired resistance ultimately occurs.^447,449^ Kurppa et al.^450^ observed that a subpopulation of cancer cells can enter a senescence-like, dormant state to escape cell death upon the administration of EGFRi (osimertinib) in combination with MEKi (tretinamib), leading to resistance (Fig. 2b). This change is regarded as a highly reversible process characterized by YAP/TEAD-mediated activation of EMT or MET programs.^451^ As expected, the therapeutic strategy of pharmacologically cotargeting YAP/TEAD (by MYF-01-37) and EGFR/MEK leads to synthetic lethality, the realization of which predominantly depends on the phenotypic switching from the dormant/senescence, mesenchymal-like, EGFRi/MEKi-refractory state to a proliferative, epithelial-like, EGFRi/MEKi-susceptible state of *EGFR*-mutant NSCLC^450,451^ (Fig. 3b). Analogously, in the context of resistance to KRAS suppression, Shao et al.^250^ also found that increased activity of YAP helps bypass loss of KRAS signaling, which at least in part depends on EMT programming. Taken together, this phenotype switching between actively proliferative and senescence-like dormant state may reflect one particular manifestation of EMT programs under drug exposure, which acts in a YAP-dependent manner and serves as an adaptation mechanism against loss of oncogene (EGFR and KRAS) signaling.^250,450,451^

#### EMT-targeted compounds in clinical trials

As apparent from the above discussion, targeting the EMT process or cell plasticity holds great potential for circumventing drug resistance. However, only a few compounds specifically designed to inhibit the EMT process are currently in clinical trial. Specifically, AB-16B5 is an antibody which directly against secreted clusterin, a stimulator of EMT programming and subsequent cancer progression.^297,452^ AB-16B5 is now being evaluated in a phase I clinical trial in advanced solid malignancy. Moreover, AB-16B5 combined with docetaxel is being evaluated in subjects with metastatic NSCLC (Clinicaltrials.gov identifier NCT04364620). Inhibitors targeting Notch, TGF-β, and Wnt signaling pathways are also promising candidates for suppressing EMT and cell plasticity. For example, PF-03446962 and galunisertib are antagonists designed to inhibit the TGF-β receptor and are currently in phase I clinical studies on solid cancers (Clinicaltrials.gov identifier NCT00557856, NCT02423343). Both PF-03446962 and galunisertib can inhibit the EMT programs and thus impede cancer development.^453,454^ In addition, Wnt inhibitors such as ETC-1922159 and OMP-54F28 have been reported to inhibit the EMT programs and are now in phase I clinical trial in solid cancers (Clinicaltrials.gov identifier NCT02521844, NCT01608867). ETC-1922159 can inhibit the secretion of Wnt ligands, while OMP-54F28 is a recombinant protein that directly binds to Wnt ligands, and thus blocks Wnt signaling effectively and its resulting cancer progression, such as those related to EMT programs.

## Transition between non-CSC and CSC states

### Definition and characteristics of CSCs

The emergence of CSCs relies on the existence of rare, immature subpopulations of tumorigenic cells within solid tumors or hematological malignancies that display the potential of indefinite proliferation and clonal long-term repopulation, along with the capability of self-renewal and differentiation (the defining features of a CSC), which contributes to tumor initiation and heterogeneity.^455–458^ The features summarized above are responsible for a number of clinical observations with regard to CSCs, including the frequent tumor recurrence after initially effective therapies, the emergence of a tumor dormancy state, and distant metastasis.^458–460^ It is known that CSCs share considerable commonalities with adult stem cells, such as identical surface markers (ALDH1, CD133, and CD44), re-activation of development-related pathways, and lack of differentiation.^461^ It is worth mentioning here that, similar to the normal stem cell that can give rise to a new stem cell with a committed progenitor, CSCs possess the ability of asymmetrical mitosis, a process regulated by multiple intricate mechanisms, yielding one daughter cell that remains as a CSC to sustain its self-renewal potential, and a progenitor or committed cell (transient amplifying [TA] cell), which is equipped with a high proliferative capability committed to differentiating into non-CSCs, constituting the bulk of the tumor.^455,461,462^ In addition to asymmetric division, symmetric division, which is characterized by the fact that daughter cells derived from a CSC appear to be either two CSCs (symmetric renewal) or two stem-committed cells (symmetric commitment), can also exist simultaneously.^463,464^ In light of these two modes of division, it is important to note that TA cells generated from asymmetric division, under certain circumstances (e.g., anti-CSCs therapy, or CSC loss), are more inclined towards dedifferentiation rather than differentiation, which allows their re-acquisition of a stem cell-like phenotype, serving to “replenish” the CSC pool on an inexhaustible basis.^464,465^ Indeed, such a functional plasticity of TA cells between “forming a tumor” and “recharging a CSC pool” is essentially a bidirectional and hierarchical plasticity of CSC regulation endowed by asymmetric division, through which a dynamic equilibrium between CSCs and non-CSCs (i.e., dedifferentiation and differentiation, or dormancy and proliferation) can be maintained.^464,465^ Intriguingly, two biological events both relevant to cellular plasticity (the EMT programs mentioned above and CSC division) are not in parallel but in tandem; or more specifically, EMT can regulate the process of CSC division in a desired orientation depending on certain scenarios. For example, several lines of evidence show that some EMT-TFs (i.e., Twist2 and Snail) are able to elicit robust regulation of stemness traits in lung CSCs by augmenting symmetric division, yet repressing asymmetric division.^466^ Conversely, in another recent study, EMT seems to play a role in maintaining and enhancing a stem cell-like phenotype by directing breast CSCs towards asymmetric division.^467^ Together, these conflicting findings regarding the links between EMT programs and CSC divisional profiling could induce a pattern of plasticity adjustment in specific types of cancer, where CSCs tend towards either symmetric or asymmetric division with EMT-TF involvement. This is a manifestation of cell fate plasticity, leading to alterations on the proportion and composition of different phenotypic subtypes within an individual tumor.

Although it is difficult to distinguish CSCs from non-CSCs, particularly special cancer types with a relative shallow hierarchy, identification of CSCs with various frequencies has been obtained using combinations of multiple cell-surface antigens in a variety of carcinomas, including leukemia,^468^ breast cancer,^469,470^ PCa,^471,472^ colorectal cancer,^473,474^ melanomas,^475^ and brain cancer.^476^ Accumulating reports also reveal that certain cancer cells can exhibit plasticity via a reversible transitioning between the CSC and non-CSC state, which repopulates the CSC pool and enables the cells to survive therapy.^49,390,477^ Specifically, in comparison to the non-CSC state, CSCs acquire resistance via several alternate mechanisms, including the upregulation of multidrug efflux pump, elevated DNA-repair capacity, improved adaption to reactive oxygen species, maintenance of a slow-cycling state, and higher trans-differentiation potential.^478,479^ All the above-mentioned molecular mechanisms, except for trans-differentiation, have been elegantly discussed in recent reviews.^458,459,480^ We will therefore focus on CSC trans-differentiation (another type of cell plasticity) and the latest observations on potential pharmacological intervention.

### The role of CSCs trans-differentiation in drug resistance

Lineage plasticity, also known as trans-differentiation or lineage switching, is the process by which cells acquire phenotypic characteristics of another cell lineage, occurring during the process of physiological regeneration of damaged tissue.^481,482^ In the context of cancer therapy, tumor cells can transdifferentiate from a cell type dependent on the drug target to a specialized cell type that is not.^483,484^ For example, using varying types of conditioned medium, melanoma spheroid cells (CSCs) can transdifferentiate into multiple cell lineages, such as melanocytes, adipocytes, chondrocytes, or osteocytes.^485^ It has been reported that a similar trans-differentiation process could be induced by treating melanoma CSCs with unsaturated fatty acids, or by upregulation of peroxisomal proliferator receptor-γ (PPARγ).^486,487^ In line with these observations for melanoma, PPARγ agonists, as represented by the antidiabetic drug, rosiglitazone,^488^ have also been found to induce cellular re-differentiation in a variety of malignancies, including myxoid liposarcomas,^489,490^ GBM,^491^ breast cancer,^492^ and chronic myeloid leukemia.^493^

**Fig. 4:**
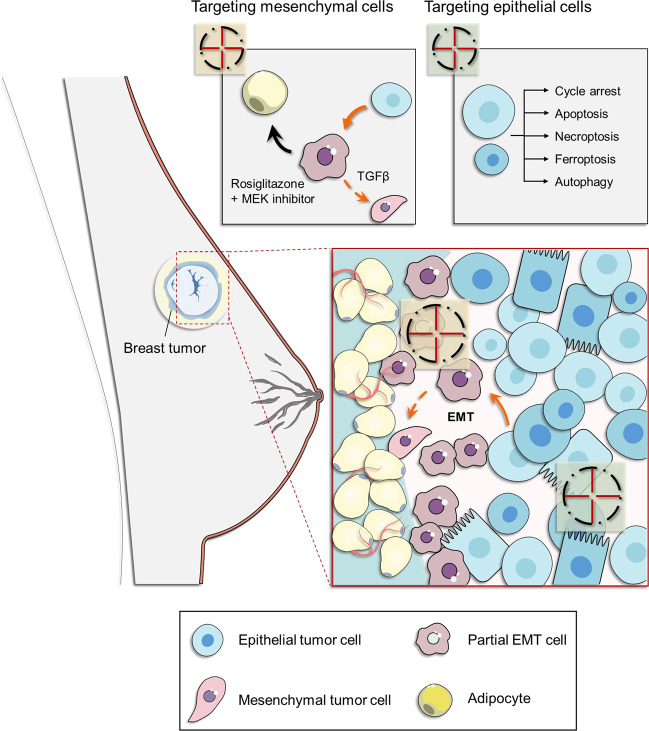
EMT can be hijacked for therapeutic purposes by forcing trans-differentiation. EMT frequently occurs at the invasive front of the individual tumor, which also allows cancer cells to achieve a high plasticity level due to the mechanistic correlation and functional overlap between the EMT process and the CSC phenotype. The mesenchymal characteristics of those tumor cells are embodied in their potential for re-differentiation and possibly even trans-differentiation. Meanwhile, cancer cells also achieve resistance to a variety of conventional therapeutics during the EMT process, commonly resulting in tumor recurrence. However, the plasticity of those cancer cells can be utilized for therapeutic purposes by forcing their trans-differentiation process towards postmitotic and well-differentiated phenotypes rather than by direct killing. The treatment of an MEK inhibitor—trametinib—together with an adipogenesis inducer—rosiglitazone—can strongly promote the direct lineage conversion of those aggressive cancer cells to “peaceful” adipocytes. This provides the potential of preventing treatment failure by combining trans-differentiation therapy with multiple conventional therapies, efficiently killing the proliferative cancer cells that form the bulk of the tumor as well as eradicating invasive cells that escape conventional therapies by the development of an EMT/CSC phenotype. EMT epithelial–mesenchymal transition, CSC cancer stem cell

Interestingly, on the basis of a well-established adipogenesis induction protocol,^494,495^ the recent research advances achieved by Ishay-Ronen et al. have provided strong evidence, indicating that the plasticity of carcinoma cells, in this case mesenchymal-like breast cancer cells (e.g., MT▵Ecad^496^ and Py2T-LT cells,^496^) can be hijacked and exploited for therapeutic purposes by forcing their trans-differentiation process towards postmitotic and functional adipocytes both in vitro and in vivo, rather than by killing directly^497–499^ (Fig. 4). Of note, this unpredicted trans-differentiation—from cancer to fat, only occurs in cell lines with mesenchymal features rather than those with epithelial features (e.g., MTflECad^500^ and Py2T cells.^496^) This was confirmed^497^ using specific markers or stains for different stages during adipogenesis, such as C/EBPα (CCAAT/enhancer-binding protein α) for preadipocytes,^501^ and Nile Red for lipid droplets.^502^ Such findings, to a certain degree, mirror the results from previous studies, which have concluded that EMT-derived cells, akin to MSCs, are equipped with the potential capacity of multilineage trans-differentiation, particularly the three mesodermal lineages: osteoblasts, chondrocytes, and adipocytes.^503–506^ This supports the notion that carcinoma cells must meet the explicit precondition of achieving a high plasticity level, and then sustaining its superiority, prior to trans-differentiation being implemented endogenously or exogenously. To put this in context, the superiority of stronger plasticity of tumor cells with mesenchymal attributes (e.g., those exhibiting partial/intermediate EMT programs) is not embodied in their increased capability for invading the surrounding stroma, but in their enormous potential for trans- and re-differentiation due to the mechanistic connection and functional overlap between EMT process and the CSC phenotype^55,497–499,503,507^ (Fig. 4). Further analysis has found that TGF-β—key promoter and sustainer of EMT programming,^165,166^ which is also known for its negative role in adipocyte development^508,509^ represses the adipogenic trans-differentiation of EMT-derived breast cancer cells by activating non-canonical MEK/ERK signaling pathways.^497–499^ As predicted, the combination therapy of an MEKi (trametinib) with an adipogenesis inducer (rosiglitazone), termed adipogenesis therapy or trans-differentiation therapy, strongly promotes the direct lineage conversion of those tumor cells that transdifferentiate from an “aggressive” mesenchymal phenotype to “peaceful” adipocytes in a PDX model^497–499^ (Fig. 4). This promotion, however, has proven to be strictly limited to cancer cells with strengthened plasticity, more specifically to those at the invasive front of the primary tumor, which is hypothesized to be the region where EMT most frequently occurs^77^ (Fig. 4). This so-called “limitation” of adipogenesis therapy, manifesting itself as spatial and functional specificity, is also a “benefit” precisely because those adipogenesis therapy-targeted cells are intrinsically more refractory to existing therapeutic approaches due to the closely mechanistic link between EMT, CSCs, and drug resistance (as mentioned earlier in this review and discussed comprehensively by Shibue and Weinberg.^55^) (Fig. 4). This suggests the possibility of avoiding treatment failures by trans-differentiation therapy alone or in combination with multiple conventional therapies, including chemotherapy, targeted therapy, radiotherapy, or immunotherapy^497–499^ (Fig. 4). The combination of conventional therapy and trans-differentiation therapy likened to the proverbial “killing two birds with one stone” where the conventional therapies efficiently kill the proliferative cancer cells that form the bulk of the tumor,^510,511^ while trans-differentiation therapy^497^ eradicates invasive cells in areas of tumor budding that escape conventional therapies by the development of a dedifferentiated EMT/CSC phenotype^497–499^ (Fig. 4). However, this promising trans-differentiation-based strategy still needs to be further validated, refined, and extended from current preclinical proof-of-concept trials to a proven clinical demonstration of successful breast cancer treatment.

In the clinical setting, it is worth contemplating all-*trans* retinoic acid (ATRA)-based regimens for treating acute promyelocytic leukemia (APL), during which accumulating abnormal promyelocytes break the differentiation block by both a change in configuration as well as degradation of the PML-RARα (promyelocytic leukemia-retinoic acid receptor α), the oncogenic fusion protein in APL pathogenesis, leading to mature granulocytes.^512–516^ This has significant clinical benefits with ~85% CR.^512–514^ Although by definition there are differences between ATRA-based differentiation therapy and adipogenesis-based trans-differentiation therapy,^484,517,518^ it is undeniable that APL cells share a great deal in common with EMT-derived carcinoma cells. Specifically, high plasticity and stem cell-like (re-)differentiation capability either empower tumor cells themselves with a spontaneous drug-refractory phenotype or render them vulnerable to a terminal differentiation state through appropriate pharmacological interventions. However, in current practice, progressive resistance to monotherapy with ATRA emerges over a short period, typically within 3–6 months.^512–514^ Adipogenesis therapy could be the beginning of novel trans-differentiation-based therapies yet to be developed.

The above examples would suggest that the activation of cancer cell plasticity by driving CSCs (trans-)differentiation could be a promising therapeutic approach for overcoming drug resistance. However, “opportunities” frequently come with “risks”. On the one hand, considering the close correlation between adipose tissue and breast cancer, the adipocyte­-rich TME resulting from trans-differentiation therapy has an increased risk of further supporting the growth and metastasis of residual cancer cells, functioning as so-called cancer­-associated adipocytes.^519,520^ On the other hand, trans-differentiation, particularly neuroendocrine trans-differentiation, has frequently been linked to disturbing side effects, including aggressiveness and resistance,^54,521^ warning that hijacking such a process for therapeutic purposes will not be without risk. Some examples are given below.

### Neuroendocrine trans-differentiation from CRPC to NEPC

**Fig. 5:**
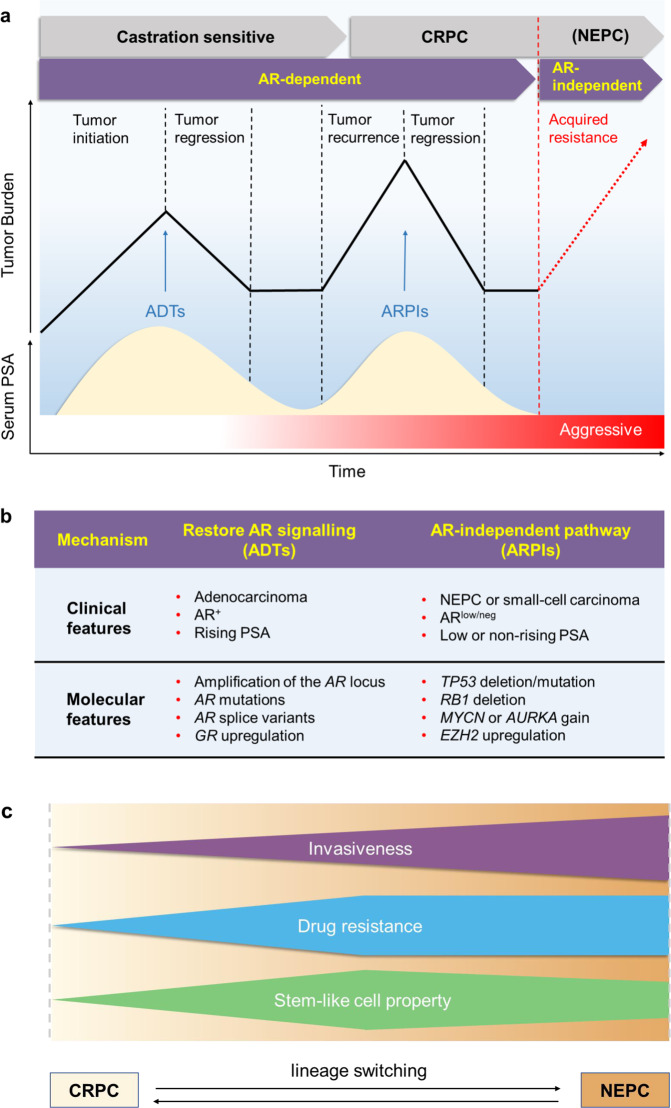
Trans-differentiation from castration-sensitive PCa to CRPC to NEPC: involvement of two generations of AR pathway-targeted agent. **a** ADTs mediate CRPC generation in an AR-independent manner, while ARPIs trigger NEPC formation dependent on AR signaling. ADTs blocking AR-related signaling, which exhibit remarkable activity causing tumor regression, lead to the emergence of aggressive CRPC with tumor recurrence. Likewise, the novel ARPIs by targeting specific ADT resistance contribute to tumor regression, whereas inducing a more aggressive NEPC phenotype in the process of neuroendocrine trans-differentiation promotes later acquisition of therapy resistance. **b** The characteristics of lineage switching from CRPC to NEPC in terms of clinical histology and molecular levels. Alteration of cellular identity from CRPC to NEPC is mainly characterized by the absence of AR and PSA. NEPC is also different from CRPC due to deletion of *TP53* and *RB1*, enhancement of MYCN or AURKA, and upregulation of EZH2. **c** Aggressive behavior accompanying functional transformation from CRPC to NEPC. As lineage switching occurs, CRPC is converted to NEPC accompanied by increased invasiveness, intensive drug resistance, and elevated stem-like cell properties. CRPC castration-resistant prostate cancer, NEPC neuroendocrine prostate cancer, AR androgen receptor, ADTs androgen deprivation therapies, ARPIs AR pathway inhibitors, PSA serum prostate-specific antigen

Although trans-differentiation of CSCs may offer a novel therapeutic avenue, drug-induced neuroendocrine trans-differentiation observed in PCa and NSCLC shows evidence to the contrary.^54,521^ PCa can be taken as a convincing example. PCa, a hormone-dependent cancer, is characterized by the high dependence of AR-related signaling for tumor growth and survival during the early stages.^522^ Treatment advantage can be taken made of this dependence. Current first-line therapy, based on ADTs and targeting the AR itself, has already been proven to be effective clinically for the prevention of PCa growth^523^ (Fig. 5a). Unfortunately, this effect is often limited, ranging from months to a few years.^524,525^ This is mainly due to the potential of PCa cells to adjust to ADTs, reflected in the re-activation of AR-mediated signaling through various mechanisms, including functional residual androgens, genomic amplification of the *AR* locus, *AR* ligand-binding domain mutations, and *AR* splice variants.^54,524,525^ All these result in the same outcome: a more aggressive form of PCa known as CRPC^526^ (Fig. 5).

However, even at this stage, there is a dependence on AR signaling.^524–526^ This has given rise to the emergence of second-generation AR pathway inhibitors (ARPIs), which act by targeting the specific ADT-resistant mechanisms by which AR signaling can be re-activated.^525–528^ As expected, satisfactory clinical therapeutic effects have been achieved with ARPIs (abiraterone acetate [abiraterone] and enzalutamide) in patients with CRPC^529,530^ (Fig. 5a). Despite this, new resistance to ARPIs inevitably occurs.^54,525^ Mechanistically, unlike the process of resistance to ADTs, emerging evidence suggests that resistance to ARPIs tends to be developed in an AR-independent manner by significantly altering the typical course of CRPC, including lineage conversion (neuroendocrine trans-differentiation) and/or the induction of EMT programs^26,54,525^ (Fig. 5). The former is termed as therapy-induced lineage crisis.^531^ Histologically, such a cellular identity crisis manifests as lineage switching from CRPC to neuroendocrine prostate cancer (NEPC), which is characterized by the presence of neuroendocrine markers (chromogranin A, synaptophysin, etc.) with the absence of AR and serum prostate-specific antigen^532,533^ (Fig. 5). At the molecular level, NEPC shares similar features to primary neuroendocrine tumors, such as combined inactivation of *TP53* and *RB1*, as well as amplification of *MYCN*^54,533–535^ (Fig. 5b). Functionally, NEPC cells are associated with multiple aggressive behaviors, such as strong metastatic potential, enhanced stem cell-like (re-)differentiation capability, and heightened therapeutic resistance, all of which ultimately result in worse prognosis^54,536^ (Fig. 5c).

**Fig. 6:**
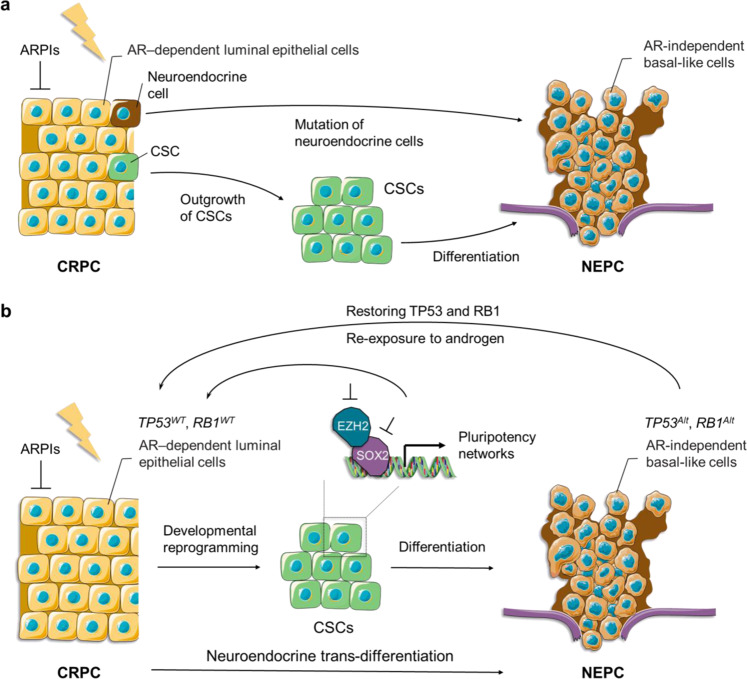
Two models describing the mechanism of lineage switching from CRPC to NEPC. **a** Following ARPI treatment, NEPC can originate from a small subpopulation of mutated neuroendocrine cells surrounding the primary tumor in CRPC, or derive from overgrown CSCs in CRPC, which undergo a differentiation process to acquire an AR-independent basal-like phenotype. **b** AR-dependent luminal epithelial cells initially undergo developmental reprogramming to neurological PCSCs, followed by differentiation into an AR-independent basal-like NEPC by ARPI-induced neuroendocrine trans-differentiation. Due to dynamic reversible cancer cell plasticity, the newly acquired NEPC can be reverted to the luminal epithelial-like CRPC by restoring *TP53* and *RB1*, re-exposure to androgen or inhibition of EZH2 and SOX2 implicated in pluripotency networks. PCSCs prostate cancer stem cells, SOX2 SRY-box transcription factor 2, EZH2 enhancer of zeste homolog 2

The precise mechanisms by which ARPIs induce lineage crisis in CRPC still remain to be elucidated. Despite that imperfect understanding, a large body of evidence suggests that lineage plasticity of tumor cell may act as a plausible explanation for the emergence of NEPC and ARPIs resistance.^26,54^ There also exists a possibility that NEPC could originate from a small subpopulation of neuroendocrine cells surrounding the primary tumor without accompanying lineage conversion,^537^ which needs to be clarified (Fig. 6a). Proceeding from that, from the analysis of clinical data, the frequency of PCa-specific genetic alterations—represented by *TMPRSS2*-*ERG* gene rearrangement^538^ in castration-sensitive prostate cancer (CSPC), has been shown to be similar to that of NEPC (46%, 45% respectively).^539,540^ This coincidence helps to confirm that late-stage NEPC is derived from early-stage CSPC.^533^ Further evidence has been gathered from an investigation on divergent clonal evolution, which identified that CRPC and NEPC share essential genetic alterations, not limited to *TMPRSS2*-*ERG* fusions, with each other.^534^ According to the above clinical and laboratory findings, it can be safely concluded that, in most cases, the emergence of NEPC under ARPIs treatment is the consequence of lineage plasticity, also termed as neuroendocrine trans-differentiation. Second, ADT-induced neuroendocrine trans-differentiation initially requires developmental reprogramming to prostate cancer stem cells (PCSCs) of a neural class in AR-dependent cell lines^541–543^ (Fig. 6b). Such reprogrammed cells lose their features of prostate differentiation becoming neural/neural crest stem cells, resulting in a malignant phenotype resistant to ARPIs in vitro and in vivo^543^ (Fig. 6b). Further research performed in genetically engineered mouse models (GEMM) showed that the resistance acquired by lineage trans-differentiation from AR-dependent CRPC to AR-independent NEPC is largely reliant on the upregulation of the well-established undifferentiated cell marker SOX2 (SRY-Box Transcription Factor 2) and epigenetic regulator EZH2 (histone methyltransferase enhancer of zeste homolog 2)^544,545^ (Fig. 6b). Given the reversibility of tumor cell plasticity, such a basal-like NEPC can partially be reverted to its original lineage—luminal epithelial-like CRPC, by the re-establishment of *TP53* and *RB1* biological functions, re-exposure to androgens or inhibition of *SOX2* expression^543–545^ (Fig. 6b). Notably, TF SOX2 is shown to play an important role in embryonic development and transcription modification, which is indeed implicated in the acquisition and maintenance of stem-like cell properties, especially for the central nervous system.^546,547^ In addition, a model of MUC1-driven lineage plasticity in PCa shows that MUC1 can upregulate the expression of *BRN2* by recruitment of MYC and subsequent binding to the promoter region of *BRN2*, which further contributes to SOX2 expression.^548,549^ This series of molecular regulations indicates that MUC1 may act as an upstream effector to regulate SOX2-induced lineage plasticity of neuroendocrine trans-differentiation in PCa.^548^ Finally, by integrating systemic analyses of GEMM with patient clinical data, Zou et al.^550^ provided conclusive genetic evidence that drug-induced neuroendocrine trans-differentiation of PCSCs is one of the main reasons behind treatment failure.^551^ Taken together, findings from different approaches, including clinical data, together with multiple in vitro and in vivo experimental models, strongly demonstrate that tumor cell plasticity-induced lineage switching enables PCa to escape from ARPI treatment.^543–545,548–551^

#### Neuroendocrine trans-differentiation from NSCLC to SCLC

**Fig. 7:**
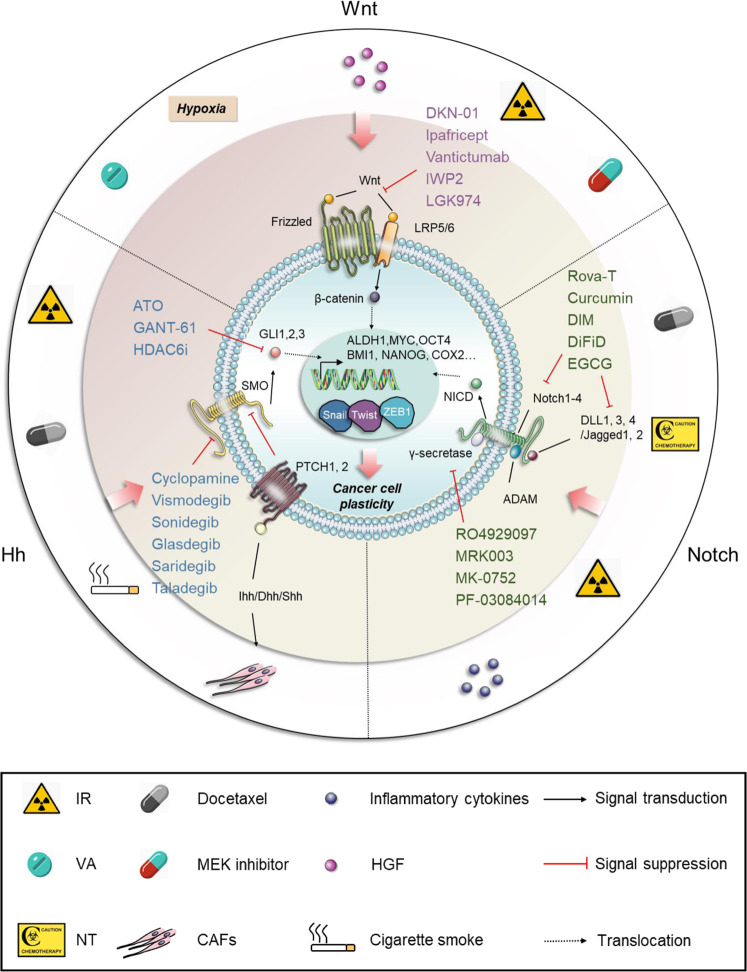
Overview of the molecular basis of re-activation of developmental programs contributing to cancer cell plasticity—major inhibitors of Hh, Wnt, and Notch signaling pathways for targeted therapy. The Hedgehog (Hh), wingless/integrated (Wnt), and Notch signaling pathways play a crucial part in acquisition and expansion of CSC phenotype after being stimulated by internal factors or extrinsic stimuli, for instance, HGF and docetaxel. Re-activation of these developmental programs promotes the corresponding transcription factors entering into the nucleus to regulate expression of downstream effectors that are closely related to CSCs regeneration and maintenance, as well as multiple biological functions. To prevent cancer cell plasticity, particularly phenotypic switching from non-CSC to CSC states induced by Hh, Wnt, and Notch signaling pathways, a range of inhibitors targeting these pathways have been approved for clinical use or are under development, as shown in blue, purple, and green. CAFs cancer-associated fibroblasts, Ihh/Dhh/Shh: Sonic hedgehog/Indian hedgehog/Desert hedgehog, PTCH1 2 patched1 and patched2, SMO smoothened, ATO arsenic trioxide, HDAC6i histone deacetylase 6 inhibitor, GLI glioma-associated oncogene homolog, LRP5/6 lipoprotein receptor-related protein 5/6, HGF hepatocyte growth factor, VA valproic acid, EMT epithelial–mesenchymal transition, NICD Notch intracellular region, ADAM a disintegrin and metalloprotease, DLL1, 3, 4 Delta-like ligand 1, 3, 4, Jagged 1, 2 Serrate-like ligand 1, 2, IR ionizing radiation, NT neoadjuvant therapy, Rova-T rovalpituzumab tesirine, DIM 3,3′-diindolylmethane, DiFiD 3,5-bis (2,4-difluorobenzylidene)-4-piperidone, EGCG epigallocatechin-3-gallate

Similar observations of neuroendocrine trans-differentiation have been made for lung cancer, in which the transition from EGFR-mutant NSCLC to small cell lung cancer (SCLC) (Fig. 7), the most thoroughly described example of lineage plasticity to date, is closely correlated to resistance to EGFR-TKIs.^26,521,552^ SCLC is a highly aggressive neuroendocrine tumor with characteristics of neuroendocrine organ-like nest-like structure, a rapid growth rate, and a natural propensity for early metastatic spread, not typically observed in patients with no history of smoking.^553–555^ The first description of this trans-differentiation was a case report showing that a 45-year-old patient diagnosed with EGFR-mutant lung adenocarcinoma had obtained a partial response to erlotinib treatment, but 18 months later relapsed with metastatic synaptophysin (a typical neuroendocrine marker)-positive SCLC in multiple organs, with no trace of adenocarcinoma at autopsy.^556^ The contributions made by neuroendocrine trans-differentiation to the resistance to TKI-based therapy in EGFR-mutant NSCLC have been excellently presented in a number of recent reviews.^26,521^

### The mechanism behind re-activation of developmental programs

Although somatic and neoplastic stem cells reside at the top of the lineage hierarchy, extensive studies have shown that, under certain conditions, not all carcinoma types strictly conform to the generally accepted unidirectional hierarchical model of CSC. This results in a phenotypic switching whereby non-CSCs acquire characteristics of CSCs, such as self-renewal capacity and a slow-cycling state.^557–559^ This reversible plasticity of carcinoma cells, a bidirectional interconversion between CSC and non-CSC states, is central to its role in tumor progression and prognosis of individual cancer patients, given the extensive evidence that CSCs are intrinsically more prone to disseminating to distant organs and are, at the same time, more refractory to existing antitumor therapies. Further in-depth understanding of this switching process is clearly urgently needed.^560,561^

From a macroscopic point of view, re-activation of transcriptional cascades rewrites cell fate in response to a wide variety of signals derived from different developmental signaling pathways involved in embryonic development, tissue homeostasis, and adult diseases processes (e.g., carcinoma progression, harboring Hedgehog [Hh], Wnt, and Notch pathways).^562–564^ The (re-)activation of developmental programs can be either beneficial or harmful to organ functions, depending on whether it occurs during early development or in the adult.^562–564^ By narrowing the focus to carcinoma progression, growing evidence reveals that these evolutionarily conserved developmental pathways are re-activated during tumorigenesis and are crucial to the acquisition and expansion of the CSC phenotype by interacting with each other, or other oncogenic signaling pathways including NF-κB, MAPK, PI3K/AKT/mTOR (mammalian target of rapamycin), and EGF.^564,565^ Of note, the contributions made by the reciprocity of these complicated pathways to CSC generation and biological function can be traced to Hh/Wnt/Notch-induced fluctuation of expression of their downstream effectors in response to external or internal stimuli. These include various cytokines and growth factors, and markers of apoptosis/antiapoptosis, proliferation, metastasis, and resistance.^566^

The above suggests that cancer cell plasticity induced by the re-activation of developmental programs to some extent determines the organizational structure and highly heterogeneous nature of individual tumors, arising from cancer cells in varying stages of differentiation. This highlights the concept that CSCs, except for those that are pre-existing, can be generated de novo from bulk non-CSCs at a low but non-negligible rate. More importantly, this provides biological insight into the therapeutic potential of targeting these pathways.^567^ Undoubtedly, this bidirectional phenotypic transition between non-CSC and CSC states has brought enormous challenge for effective clinical treatment and is driving a marked shift of attention towards the optimization of developmental pathway-targeted therapy.^568^ Details on the relationship between the acquisition and maintenance of CSC phenotype and CSC-dependent signaling pathways, as well as the development of appropriate Hh/Wnt/Notch signal-targeted drugs for preclinical or clinical trials will be discussed below.

#### Hh pathway

The Hh signaling pathway is intimately concerned with cell proliferation and differentiation, together with tissue homeostasis and regeneration throughout life. However, aberrant activation of Hh has also made it a force to be reckoned with in oncology due to its involvement in tumorigenesis and progression, from the formation of tumor-initiating cells to angiogenesis as well as tumor immune escape.^569,570^ It is known that Hh signal transmission is largely managed by two multitransmembrane receptors on the target cell: Patched (PTCH1 and PTCH2) and Smoothened (SMO): the former is characterized by antagonizing effects, while the latter has positive regulatory functions.^571^ When levels of secreted Hh ligands (Sonic hedgehog [SHH]/Indian hedgehog/Desert hedgehog) are low or non-existent, the transmembrane receptor PTCH is stably situated in the primary cilium (PC), thereby inhibiting SMO activity and preventing further signal transduction.^572^ However, in the presence of Hh ligands, PTCH can dissociate from the PC and simultaneously relieve its repressive effects on SMO following binding by extracellular Hh ligands. This in turn facilitates signal transmission and consequent activation and nuclear localization of the Hh pathway downstream effector GLI (glioma-associated oncogene homolog, GLI1, GLI2, and GLI3) accompanied by upregulated expression of Hh target genes participating in differentiation, proliferation, and survival.^563,565,571,573^

Deregulation of the Hh pathway occurs in diverse cancers, including those of the breast, lung, bladder, pancreas, and stomach.^574–578^ Since there exists closely functional overlap between EMT programs and the CSC phenotype,^55^ the associated signaling pathways involved in sustaining the mesenchymal (or quasi-mesenchymal) state of carcinoma cells in various tissues has been a major topic of research. In particular, radiotherapy has been found to stimulate re-activation of Hh signaling, which further induces the EMT process by overexpression of EMT-stimulating factors and mesenchymal markers.^579^

Studies on multiple myeloma have suggested that Hh signaling is involved in interactions between CSCs, differentiated cancer cells, and the microenvironment, whereby blocking signaling can result in CSC differentiation.^580^ In TNBC, the acquisition of a chemo-resistant and stem-like phenotype benefits from a supportive niche with expression of fibroblast growth factor 5 and production of fibrillar collagen, which is provided by CAFs that are reprogrammed by newly secreted Hh ligands.^581^ The administration of docetaxel leads to the release of SHH ligand, followed by activation of the Hh pathway. Furthermore, increases in the expression of stemness signature with breast mammosphere formation provide information on the connection between chemotherapy-induced Hh signaling and expansion of breast CSC populations.^582^ Intriguingly, in kidney cancer, cigarette smoke triggers the activation of the SHH pathway, thus enhancing tumorsphere formation and elevating renal CSC populations. This finding from a series of experiments supports a molecular mechanism of cigarette smoke-elicited stemness by Hh signaling activation.^583^

Aberrations in Hh cascade contributing to tumorigenesis and tumor progress indicate that the Hh pathway represents a valid target for cancer therapy clinically. In particular, drugs targeting SMO have attracted considerable interest. Cyclopamine, an alkaloid extracted from *Veratrum californium*, was the first identified Hh inhibitor suppressing CSC proliferation with effective control of Hh-dependent tumors.^584,585^ As a first-in-class, the cyclopamine-competitive SMO antagonist, vismodegib, is effective in reducing the content and/or viability of breast CSCs. It was licensed for the treatment of metastatic basal cell carcinoma by the US Food and Drug Administration (FDA) in 2012 and the European Medicines Agency in 2013.^586,587^ There is also considerable interest in the selective SMO antagonist sonidegib due to its success in the treatment of advanced basal cell carcinoma, resulting in its launch in the USA in 2015.^568,588^ Other selective SMO inhibitors (e.g., glasdegib, saridegib, and taladegib) have entered a number of clinical trials, including metastatic or recurrent head and neck squamous cell carcinoma, and acute myeloid leukemia.^589–591^ Regrettably, a number of approved SMO inhibitors that serve as single-target agents display a ubiquitous toxic reaction and build up chemotherapy resistance, indicating that further development of Hh signaling pathway inhibitors is required to overcome these common side effects.

SMO-dependent activation of GLI TFs, a late stage of the Hh pathway that regulates the expression of critical developmental genes, is another possible target.^592^ Arsenic trioxide (ATO), an FDA-approved drug which directly binds to GLI1 and GLI2, is highly effective in inhibiting Hh signaling, further causing induction of differentiation and apoptosis of CSCs.^593^ This results in higher remission rates and significantly longer survival in APL.^593^ Analogously, GANT-61 is another type of GLI inhibitor, currently under preclinical study, which is capable of preventing DNA binding to GLI1 and GLI2.^594^ HDAC6 inhibitor, which can promote differentiation and decrease the stemness of glioma stem cells via inactivation of SHH/GLI1 signaling, is another drug that can overcome stemness by targeting that pathway.^595^

#### Wnt pathway

Generally, during embryonic development, extracellular Wnt proteins monitor and modulate a variety of cellular processes, including cell proliferation and differentiation, while in adulthood Wnt signaling participates in the maintenance of somatic stem cell identity and orientation differentiation of MSCs.^596,597^ In brief, intracellular Wnt signaling functions through an autocrine or paracrine mode, either by the canonical pathway (Wnt/β-catenin pathway) or non-canonical pathways (the planar cell polarity pathway, which involves jun N-terminal kinase and the Wnt/Ca^2+^ pathway).^596,598^ The β-catenin-dependent Wnt pathway is highly conserved through evolution and is activated by interactions between Wnt proteins and their respective receptors, the seven-transmembrane receptor Frizzled (FZD) and the single-pass, low-density lipoprotein receptor-related proteins 5 or 6.^599,600^ In addition, in the case of Wnt signal, β-catenin accumulates in the cytoplasm and then localizes in the nucleus instead of being ubiquitinated and degraded, thus driving the transcription of the stemness-related target genes and inducing a series of cellular reactions.^601^

Much of the research on CSC characteristics has examined whether Wnt signaling is crucially tethered to EMT with acquisition of stem-like properties.^135^ It is known that Wnt signaling can stabilize β-catenin proteins along with the typical EMT marker—Snail, in a tandem fashion and generate TCF/LEF (T cytokine/lymphocyte enhancer) transcriptional machinery so as to cooperatively govern EMT, thereby initiating tumor cell dedifferentiation.^602,603^ It has also been postulated that Wnt5 signals via the FZD2 receptor and FYN (an Src family kinase) activate STAT3 transcription to trigger EMT programming through the previously unrecognized, Wnt5-FZD2 non-canonical pathway. This has been observed in multiple cancer cell lines as well as a mouse xenograft model.^604,605^ Wnt/β-catenin signaling has been discovered to affect EMT stimulated by ionizing radiation (IR), whereby upregulation of Wnt ligand and nuclear accumulation of β-catenin with elevated β-catenin/T cell factor transcriptional activities can be induced by IR.^606–608^ IR-induced Wnt/β-catenin signaling expedites activation of EMT by enhancing Snail protein stability.^609^ In the case of radioresistance, ribosomal S6 protein kinase 4, which has been reported to contribute to therapeutic resistance and poor prognosis, phosphorylates GSK-3β directly at Ser9, activating the Wnt/β-catenin pathway and acquiring CSC properties in esophageal squamous cell carcinoma.^610^ It is worth mentioning that Wnt signal can be coupled with Notch to induce a liver CSC phenotype; there appears to be a decrease in expression levels of a number of TFs implicated in EMT with a loss of CSC properties like self-renewal and tumorigenicity when Wnt or Notch signaling is blocked.^611^

Besides intrinsic factors, a dynamic shift from a differentiated to a stemness state of cancer cells can occur in response to extrinsic environmental cues.^612^ Consistent with this notion, hepatocyte growth factor, a myofibroblast-secreted factor, assists colorectal cancer cells to attain a stemness-like state from a differentiated, mature phenotype by β-catenin-dependent transcription both in vitro and in vivo.^612^ A study utilizing patient-derived colorectal cancer organoids has demonstrated that clinical use of MEKi (selumetinib, trametinib, and PD318088) unfortunately enhances Wnt activity and enrichment of gene signatures of stemness and relapse, ultimately inducing cancer cell plasticity.^613^ Likewise, valproic acid, used as an HDAC inhibitor and an anticancer agent in breast cancer clinical trials, has been found to be responsible for the upregulation of Wnt reporter activity, which enlarges the breast CSC pool through dedifferentiation of non-stem-like cells and promotes their capacity to generate tumors.^614^ Another study in B cell lymphoma concluded that once cancer cells escape from chemotherapy-induced senescence, they are much more likely to re-enter the cell cycle with strongly elevated Wnt-dependent clonogenicity as well as substantial upregulation of stem cell signatures.^615^ In human GBM and breast cancer, it has been proven that activation of TGF-β associated with Wnt pathways can induce an undifferentiated state to promote stemness under hypoxia.^165,616^

Review of the literature shows that dysregulation of the Wnt pathways exerts distinct functions in the dedifferentiation of CSCs. Once Wnt/β-catenin signaling is activated, PMP22 (peripheral myelin protein 22), an integral membrane glycoprotein, causes differentiation of gastric CSCs, whose mRNA levels decline dramatically.^617^ In contrast, tumor necrosis factor receptor-associated protein-1 inhibits the differentiation of CSCs by adjusting ubiquitination or phosphorylation of β-catenin in colorectal carcinoma.^618^

Although promising advances have been made in the development of inhibitors blocking the Hh pathway in early phase clinical trials, the development of drugs targeting the Wnt pathway still seems to face serious challenges. To date, relatively few agents have successfully reached clinical development, although DKN-01, a humanized monoclonal antibody that binds to and blocks the activity of the Dickkopf-1 protein, modulating Wnt/β-catenin signaling, is undergoing clinical trials in a wide range of cancer types.^619^ The suppressors of the Wnt signaling pathway, ipafricept and vantictumab (both first-in-class antibodies), are well tolerated in patients and reduce the abundance and frequency of CSCs in patient-derived tumor xenograft models of numerous cancer types.^620,621^

Published results of clinical trials showed that cirmtuzumab, a monoclonal antibody targets ROR1, which serves as a receptor for Wnt5a in the Wnt-planar cell polarity pathway successfully led to a reduction in dedifferentiation marker expression in chronic lymphocytic leukemia.^622^ In addition, Foxy-5, a Wnt5a-mimicking peptide in phase I study, causes activation of downstream Wnt5a signaling in colorectal, prostate cancer, and metastatic breast cancer owing to its antimetastatic activity.^623–625^ IWP2 and LGK974, small-molecule inhibitors, have been shown effective in rodent tumor models by preventing palmitoylation of Wnt ligands and targeting a Wnt-specific acyltransferase, porcupine. On the one hand, these compounds block autocrine signaling, which sustains the stem phenotypes of existing CSCs, while on the other hand, they curb paracrine signaling transmission that triggers formation of regenerative CSCs.^626,627^ A recent study has shown that Myc decoy oligodeoxynucleotide (ODN) attacks the transcription targets of Wnt/β-catenin signaling, accelerating the differentiation of simulated mouse CSC models. This suggests optimizing the Myc decoy ODN approach as a prospective strategy for differentiation therapy.^628^

#### Notch pathway

Dysregulation of the Notch pathway occurs in many cancers, including leukemia, GBM, and cancers of breast, cervix, colon, endometrium, kidney, lung, pancreas, and prostate.^629^ In mammals, there are five Notch ligands (Delta-like ligand: [DLL] 1, 3, 4 and Serrate-like ligand: Jagged 1, 2), forming a class of transmembrane proteins with conserved molecular structure. There are four Notch receptors (Notch1–4) that consist of an extracellular region, transmembrane region, and intracellular region (NICD/ICN) comprising a highly evolutionarily conserved Notch pathway, together with ligands above. In the absence of Notch signal, DNA-binding protein CSL (collective name of CBF-1, Suppressor of hairless and Lag) is bound to a co-repressor complex, which leads to repression of transcription. Binding of ligands to the extracellular domain of their receptor triggers two consecutive proteolytic cleavages: initially by ADAM (a disintegrin and metalloprotease), followed by γ-secretase, generating a soluble intracellular domain (NICD) that is transferred into the nucleus upon Notch signaling. Thus, when a ligand expressed on one cell specifically binds to a receptor on the adjacent cell, NICD together with CSL protein complex lead to the conversion from the original “synergistic inhibition complex” to a “synergistic activation complex.”^630^

As has been observed for the Wnt pathway, overwhelming evidence indicates that Notch signaling exerts a major influence on the security of a pool of stem or progenitor cells during embryonic or adult developmental programs.^631^ In addition, the Notch pathway is a fundamental master pathway closely controlling the fate of CSCs.^632^ As with the aforementioned signaling pathways, multiple evidence has been generated regarding the preternatural re-activation of Notch signaling causing acceleration of proliferation and restriction of differentiation in various cancers.^633^ The Notch pathway has also been considered to be involved in mediating resistance to chemoradiotherapy in several human malignancies.^634^ Similar to the Hh and Wnt pathways, there is considerable evidence to support the functional connection between EMT and Notch signaling by dominating central processes such as stemness generation.^635^ Moreover, EMT programs in colorectal cancer can be induced by constitutively active Notch1 by retroviral transduction that activates CD44, Slug, and Smad-3 via a cascade of other Notch receptors through induction of Jagged 1 expression.^635^ In addition, modeling the effect of inflammatory cytokines in the tumor microenvironment suggests that these cytokines are likely to stabilize a hybrid epithelial/mesenchymal phenotype and improve the frequency of CSCs by activating Notch-Jagged signaling.^636^ Experimental data have implied that Notch signaling can induce EMT programming by upregulation of Snail following irradiation.^637^ Another representative finding is that irradiation is capable of inducing de novo generation of breast CSCs relying on Notch signaling, which coincides with overexpression of the TFs as well as stem cell markers (*Oct4*, *SOX2*, *Nanog*, and *Klf4*). This reprogramming can be partially prevented by Notch inhibition.^567^

Previous research reports showed that an increase in CSC subpopulations, attributed to the activation of Notch signaling together with EMT induction, occurs in breast cancer mouse models after being treated with docetaxel.^638^ In current clinical practice, to allow optimal surgery and improved prognosis, neoadjuvant therapy (NT) is widely used in patients with locally advanced or inflammatory breast cancer. However, conjunctive chemotherapy-triggered events potentially contribute to the formation of a CSC phenotype, with higher levels of nuclear Notch and stemness markers being detected in primary breast cancers following NT.^639^ Alternatively, the interplay between Wnt and Notch signaling with other critical pathways like the Hh pathway mentioned earlier specifies the differentiation/stem states of cells.^640^ Indeed, it has been demonstrated that ectopic activation of Notch is sufficient to prompt dedifferentiation and drive tumorigenic transformation of mature adipocytes in vivo.^641^

Notably, the subsequent failure of secondary tumor growth upon re-transplantation indicates that loss of Notch results in a 50% reduction of cancer-initiating cell populations in xenograft models of esophageal adenocarcinoma cells.^642^ Another study has verified that the antitumor effects of Notch blockade assist in guiding the differentiation of liver CSCs into mature hepatocytes. This depends on the inverse process of EMT, namely, MET.^643^ In addition, enhanced miR-200b-3p reduces Notch signaling followed by a depletion of pancreatic CSC populations due to their tendency for asymmetric division. Coincidentally, the miR-34a-Numb-Notch feedback loop prevents ionizing radiation-induced EMT, blocking transformation from a differentiated state to a stem-like state in NSCLC.^644^ It should be mentioned that, among the four homologs that act as ligand-activated TFs in Notch signal transduction, in contrast to Notch3 and Notch4, there are trans-activation domains present in Notch1 and Notch2. This increases the functional complexity of Notch1 and Notch2, possibly conferring on them multiple roles in cancer biology to some extent.^645^ Among such functionalities, the possible relevance of Notch1 and Notch2 to the regulation of EMT course and CSCs has been suggested. One study indicated that there could be a latent interaction network between Notch1, HIF-1α, and GPER (an alternative ER), in which Notch1 responds to distinct microenvironmental cues (e.g., estrogen or hypoxia) in the context of the interplay of HIF-1α and GPER, thus promoting the activation of EMT programming in several cancers.^646–648^ By extension, elevated expression of ERα and subsequent estrogen effects could activate the Notch pathway through its binding to the promoter region of *Notch1*, which enhances EMT status together with basal stem-like properties of prostate cancer cells.^648^ Furthermore, hypoxia has been proved to be conducive to Snail1 transcription by the promotion of a HIF-1α/NICD synergistic interaction, in turn triggering the recruitment of NICD to the *Snail1* promoter. In this way, HIF-1α facilitates EMT programming by improving activation of Snail in a Notch-dependent manner in oral squamous cell carcinoma.^647^ Perhaps, more importantly, in terms of the collaboration between GPER and HIF-1α, estrogen appears to strengthen Notch-mediated EMT by increasing HIF-1α recruitment at the *Snail* promoter via nuclear GPER.^646^ With respect to the mechanistic links between Notch2 and CSCs or EMT, in vivo and in vitro investigations on NSCLC have shown that Notch2 plays a central role in miR-181b-mediated stemness, whereas silencing Notch2 causes a striking reduction in tumorsphere formation of NSCLC cells.^649^ In breast cancer, highly active Notch2 has been regarded as a key mediator and major contributor in fractionated radiation-induced EMT via the IL-6/JAK/STAT3 signaling axis, leading to the loss of E-cadherin and elevated N-cadherin and vimentin levels.^637^ Likewise, deregulation of miR-195-5p is likely to modulate Notch2 translation and further upregulate Notch2 expression, thereby motivating EMT in colorectal cancer cell lines.^650^ In comparison to deletion of *Notch1*, forced overexpression of *Notch2* in bladder cancer displays oncogenic effects, including EMT with its effector HES1 targeting the vimentin promoter in a Snail/Slug-dependent manner, and in addition to that, Notch2 facilitates dedifferentiation accompanied by increased CSC production in vitro and in vivo.^651^

There is accumulating evidence indicating the therapeutic potential of targeting Notch signaling for its roles in the enrichment of colon and breast CSCs.^652–654^ Clearly, the inhibition of signaling through the Notch receptors reduces the subpopulations of breast CSCs and impairs tumor-initiating capacity, indicating that targeting Notch signaling can be regarded as a potential therapeutic strategy.^655^ In this respect, two approaches to inhibiting Notch signal have been tried clinically: use of γ-secretase inhibitors (GSIs) as well as antibodies against the Notch receptor or ligand.^565^

Since γ-Secretase, a multisubunit intramembrane protein complex, plays a pivotal role in Notch signal transduction by exhibiting proteolysis, it is projected to be an effective therapeutic target in cancer.^656,657^ Based on this, GSIs are the most broadly developed Notch pathway inhibitors to date. In vitro studies have presented ample evidence that GSIs decrease CSC subpopulations and tumorsphere formation, indicating that Notch signal activation is required for CSCs stemness.^658^ RO4929097, a novel molecular inhibitor of γ-secretase, impairs colony formation in primary melanoma cell lines and affects tumor formation in human primary melanoma xenografts.^659^ Weekly oral delivery of MRK003, a cyclic sulfamide GSI, exhibits prominent inhibition of tumor growth, decreased expression of stemness markers, and efficient suppression of clonogenicity potency in brain cancer, supporting its further clinical use.^660^ According to clinical/preclinical data, treatment with other functional GSIs, such as MK-0752 and PF-03084014, can cause tumor regression or induce tumor growth arrest by targeting CSCs in breast and colorectal cancer. In liver cancer, a low dose of PF-03084014 induces tumorsphere differentiation and contributes to chemosensitization, further demonstrating its future clinical potential.^634,661,662^

The atypical Notch receptor ligand DLL3 may also provide a new practicable target for treatment of neuroendocrine carcinomas. The blockbuster drug, rovalpituzumab tesirine (Rova-T), an antibody–drug conjugate targeting the protein DLL3 on tumor cells, showed good safety and efficacy when given as a monotherapy in a phase I trial on recurrent SCLCs, particularly in individuals with high levels of DLL3.^663^ It has also been noted that natural agents downregulating Notch signal, including curcumin (from turmeric), 3,3′-diindolylmethane (found in cruciferous vegetables), 3,5-bis (2,4-difluorobenzylidene)-4-piperidone (from turmeric), and epigallocatechin-3-gallate (from tea), have been proposed as alternative strategies for cancer therapy and have successfully undergone clinical trials.^664,665^

### Targeting cell plasticity of non‑CSC and CSC transition

An issue of great concern is that a single approach aimed at merely eradicating CSCs tends to be restrictive and not comprehensive enough due to its efficacy only for low-grade cancers with the acquisition of therapeutic resistance in most cases.^464,666^ This highlights the possibility that the CSC subpopulation, along with its plasticity influenced by numerous TFs (i.e., Sox gene family), multiple signaling pathways (i.e., Wnt-β-catenin, IL-6-STAT3, and retinoid X receptor signaling pathway), and tumor microenvironment containing secreted factors and extracellular matrix, may impact on clinical trials. In combination with those signaling pathways discussed above, various other pathways interact with each other, for example, Wnt and Notch, uniting in a vast and complicated network. It is unrealistic to try and block all cancer-causing pathways in a therapeutic manner. Rather, there should be a focus on identifying and then abolishing the dominant drivers of plasticity among CSCs and nearby differentiated non-CSCs in the CSC niche in situ to assist CSC-targeted therapy.^667^ Emerging systems biology data provide a means to make it possible to explore how the various elements interact and influence one another to normalize neoplastic cells. Specific core TFs might contribute to phenotypic switching by triggering alterations in the expression of a battery of genes within the corresponding regulatory network.^668^ One other point worth emphasizing is that certain epigenetic regulators, such as EZH2 and REST (repressor element-1 silencing transcription factor), involved in differentiation to a neuroendocrine phenotype (the aforementioned trans-differentiation) and resistance to routine therapy in prostate or lung cancer apply genetic or pharmacological means to inhibit their activity, aiming to reverse this phenotypic transformation, and regenerate, or maintain the drug-susceptible state.^26^ On the basis of these discoveries, there is a trend towards the development of differentiation and normalization therapy (e.g., ATRA, tranylcypromine analogs, rosmantuzumab, and oncostatin M), and combined therapy with regimens designed to target cellular components and/or related pathways within the TME (e.g., NCT01839487, NCT02030860, and NCT01621243 [a series of clinical trials of PDAC]) rather than anti-CSC therapy alone, with the potential to increase the life expectancy of a far wider range of cancer patients.^458,669–674^

Despite these advances, in order to fully achieve CR in clinical practice, novel rationally designed therapeutic approaches developed on the basis of an in-depth understanding of CSC dynamics are urgently needed. However, it is unavoidable that plasticity-targeted therapy will also be confronted with many challenges, as patients who suffer from the same type of cancer vary considerably in their response to similar treatments, highlighting the need for a personalized/precision medicine approach. The persistence of minimal residual disease (MRD) characterized by drug-tolerant cancer cells, following cancer therapy due to various forms of phenotypic switching, requires an understanding of the intratumoral heterogeneity within individual tumors through a systematic and integrated analysis of potential plasticity-associated factors. Only in this way can an optimal, effective, and personalized therapeutic strategy be formulated.

## Conclusions

### For cancer cells: better to change than be killed

At first sight, accompanied by the development of emerging therapeutic strategies (e.g., targeted therapy and immunotherapy), coupled with a solid understanding of the genetic mutations involved, advanced or even chemo-/ radiation-resistant cancers seem to be curable clinically. However, the facts suggest otherwise. While initial clinical responses to patients with later-stage carcinomas typically appear encouraging, tumor recurrence inevitably occurs in these patients after a short-lived period of non-progression. This can be evidenced by the development of molecularly targeted therapies, *i.e.*, three generations of EGFR-TKIs, to treat *EGFR*-mutant NSCLC, the results of which still have not been able to meet clinical expectations due to the acquisition of resistance. What then is the cause of this phenomenon? It could be interpreted as a consequence of de novo mutations, or similar mechanisms, which endow tumor cells with the capability of bypassing inhibition of the targeted pathway under drug exposure. However, these explanations from the perspective of genetic alterations do not fully account for the accumulating clinical and laboratory observations, thus leading to a shift in research priority, at least in part, from mutational mechanisms to those related to non-genetic alterations.

The non-mutational process largely depends on tumor cell plasticity, which is regulated by highly integrated and complex interactions between transcriptional factors, epigenetic modulators as well as a variety of growth factors, cytokines, and chemokines released from non-neoplastic cells within the TME. The impressive ability of tumor cells to switch their identities or phenotypes is more likely a common mechanism by which they can escape treatment. It should be noted that phenotypic “change” is often accompanied by the acquisition of a more aggressive behavior, especially enhanced flexibility, mainly manifested in the processes such as EMT, transition from non-CSC to CSC, or CRPC to NEPC, which will exacerbate the difficulty of clinical treatment. Even more surprising, in most cases, tumor cells can achieve a new phenotype without losing their original properties, suggesting that phenotype switching between two functionally independent states is not strictly adhering to a binary-based “all or nothing” principle, but rather is a complicated multistage dynamic process involving several intermediate phenotypes with varying degrees of maintained biological characteristics. Alternatively, plasticity may have already existed in the “arsenal” of tumor cells prior to drug exposure and thus cancer therapy actually serves as a “trigger” to stimulate “change” to avoid cell death—better to change than be killed. Although tumor plasticity has been proven to play a key role in resistance to cancer therapy, there remain numerous questions to be answered and challenges to face.

### For treatment: only “change” can prevent “change”, and make it changeless

Given its malleable nature and consequent poor clinical outcomes, understanding the true meaning of plasticity (“change”) is fundamental to unlocking the secrets of non-mutational resistance mechanisms during cancer therapy. To deal better with the “change” of carcinoma cells, it will be necessary to change both experimental methods and treatment strategies.

Using the example of EMT described earlier in this review, the cognitive evolution of the EMT concept from a “complete” to a “partial” form, to a great extent, could be viewed as a reflection of the development of experimental techniques (i.e., from dual-colorimetric RNA-ISH to scRNA-seq to LSR-3D imaging). This suggests that the ideal approach would monitor the whole dynamic process of cancer development from one phenotype to another, at both an individual and multicellular cluster level. Only when the nature of tumor plasticity is fully understood can complete prevention be truly achieved. This is likely to be based on not only existing strategies, such as intermittent treatment and combination therapy, but also the development of new strategies, such as adipogenesis therapy, which can take advantage of the vulnerability of tumor plasticity.

Finally, knowing that tumor cell plasticity plays an important role in therapeutic resistance, the prevention of this dynamic process seems to be a necessary prerequisite for the improvement of clinical outcomes for cancer patients. This assumes that the “change” of experimental methods is conducive to increasing our understanding of the mechanisms of the phenotypic “change” in cancer cells during treatment, which in turn could accelerate the “change” of therapeutic strategies to prevent tumor cell plasticity. In essence, only “change” can prevent “change,” and make it changeless.
